# Essential Oils and Their Multifunctional Activities Against *Pseudomonas aeruginosa*: Current Evidence and Perspectives

**DOI:** 10.1002/mbo3.70339

**Published:** 2026-06-25

**Authors:** Giorgia Novello, Elisa Bona, Manuel Petroselli, Chiara Bazzano, Stefano Chiesa, Tabata Gaggero, Giorgio Gaglio, Gabriele Magnani, Alfonso Pampaloni Pasetti, Simone Repetto, Erica Rotella, Alice Topino, Emanuela Serra, Manuel Vidali, Silvia Zonca, Elisa Gamalero

**Affiliations:** ^1^ Dipartimento di Scienze e Innovazione Tecnologica (DiSIT) Università del Piemonte Orientale Alessandria Italy; ^2^ Dipartimento per lo Sviluppo Sostenibile e la Transizione Ecologica Università del Piemonte Orientale Vercelli Italy

**Keywords:** antibiotic resistance, biofilm, essential oil, human opportunistic pathogens, *Pseudomonas aeruginosa*, quorum‐sensing inhibition

## Abstract

*Pseudomonas aeruginosa* is an opportunistic pathogen responsible for acute and chronic infections and is characterized by a remarkable ability to develop antibiotic resistance. This has prompted increasing interest in alternative or complementary antimicrobial strategies, including the use of essential oils (EOs), which are complex mixtures of plant‐derived volatile compounds with documented biological activities. This review aims to summarize and discuss the current evidence on the antimicrobial, antibiofilm, and anti‐virulence activities of EOs against *P. aeruginosa*, with particular emphasis on their mechanisms of action, synergistic interactions with antibiotics, and limitation to the possible clinical applicability. A large number of studies, mainly conducted in vitro, indicate that EOs and their major constituents can impair *P. aeruginosa* viability and pathogenicity through multiple mechanisms. In addition, several EOs or purified terpene components exhibit synergistic effects when combined with conventional antibiotics, enhancing antimicrobial efficacy. However, in vivo evidence remains limited, largely restricted to topical infection models, while clinical studies in humans are currently lacking. Significant challenges related to chemical variability, toxicity, safety, standardization, and regulatory classification also emerge from the literature. Although EOs show considerable promise as antimicrobial and anti‐virulence agents against *P. aeruginosa*, their current role should be viewed primarily as adjunctive or alternative strategies rather than as standalone systemic therapies. Future progress will depend on the development of standardized and safer formulations, advanced delivery systems, and well‐designed in vivo and clinical studies.

## Graphical abstract

Essential oils show multifunctional activity against multidrug‐resistant *Pseudomonas aeruginosa* by targeting bacterial membranes, quorum sensing, biofilm formation, virulence traits, and antibiotic susceptibility. Their translational potential depends on standardized composition, optimized delivery systems, safety assessment, and clinical validation.

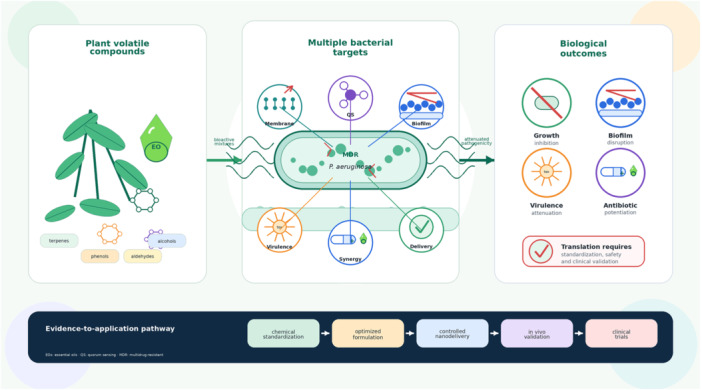

## Preface

This review was developed within the framework of an innovative teaching activity at the University of Eastern Piedmont (Università del Piemonte Orientale), Department of Science and Technological Innovation (DiSIT), as part of the Master's Degree program in Biology, Agro‐Environmental curriculum. The project invited students to actively participate in the scientific writing process by contributing to the preparation of this manuscript. This experience required considerable effort, from retrieving and critically analyzing bibliographic references to synthesizing complex information, writing in a nonnative language, and coordinating the overall structure of the review. The work was carried out under the guidance of Elisa Gamalero, Giorgia Novello, Elisa Bona, and Manuel Petroselli. We would like to sincerely thank all the students involved for their enthusiasm, commitment, and intellectual curiosity, which made this collaborative effort possible.

## 1 Introduction

### 1.1 Background on *Pseudomonas aeruginosa*

*P. aeruginosa* is a Gram‐negative opportunistic human pathogen with a surprising adaptability and an extraordinary arsenal of virulence factors that enable it to induce severe infections across humans and animals. In humans, it is one of the main causes of healthcare‐associated infections, especially among immunocompromised patients, such as those with cystic fibrosis (CF), and those exposed to invasive medical devices (Parkins et al. 2018). Besides its occurrence in the clinical context, *P. aeruginosa* is also widespread in soil and freshwater, where it can survive under nutrient‐starvation as well as in the presence of organic and inorganic pollutants (Crone et al. 2020; Hu et al. 2023). In terrestrial ecosystems, *P. aeruginosa* commonly colonizes the rhizosphere, the narrow region of soil influenced by plant root exudates, where it frequently behaves as a biocontrol agent. Its action against soil‐borne diseases is well known and mediated by the release of bioactive compounds, such as, for example, siderophores that can suppress the growth of phytopathogenic microorganisms via iron competition (Abo‐Zaid et al. 2023). This dual ecological role—as a human opportunistic pathogen and a beneficial soil inhabitant—makes *P. aeruginosa* an environmental reservoir of clinical relevance, potentially capable of transmitting resistance genes across ecological boundaries. Epidemiologically, *P. aeruginosa* continues to impose a global burden. In hospitals, it accounts for 10%–15% of all Gram‐negative infections and up to 20% of ventilator‐associated pneumonia cases (European Centre for Disease Prevention and Control [ECDC] 2024, https://www.ecdc.europa.eu/sites/default/files/documents/healthcare-associated-point-prevalence-survey-acute-care-hospitals-2022-2023.pdf). In the environment, the occurrence of *P. aeruginosa* has been detected in 55% of the agricultural soils considered (Licea‐Herrera et al. 2024).

The pathogenicity of *P. aeruginosa* is mediated by a plethora of virulence traits—including the type III secretion system, quorum‐sensing (QS)–regulated exotoxins, proteases, siderophores, pyocyanin, and the capability to form biofilms on abiotic surfaces. Adhesion to host cells is mediated by a variety of surface structures, including type IV pili and flagella, which ensure motility and adherence to epithelial cells and abiotic surfaces, such as catheters and prosthetic devices (Hinsa and O'Toole 2004). Once attached, *P. aeruginosa* can form biofilms, structured communities of bacteria embedded in an extracellular matrix of polysaccharides. Biofilm formation is typical of chronic infections, such as those in CF patients, where it provides protection against both host immune responses and antibiotic therapy (Borisova et al. 2025). These traits not only facilitate host colonization and immune evasion, but also enhance persistence in soil or on the root system, especially biofilm formation that provides a protected environment against protozoan predation and desiccation (Thi et al. 2020).

Central to *P. aeruginosa* virulence is its ability to coordinate gene expression through QS, which regulates the production of numerous virulence factors according to cell density. QS involves the use of autoinducers that, when accumulated to a certain concentration, which is proportional to cell density, activate transcription of virulence‐associated genes. Since 1994, at least four QS systems, Las (Pearson et al. 1994), Rhl (Brint and Ohman 1995), Pseudomonas Quinolone Signal (PQS) (Pesci et al. 1999), and Integrative Quorum‐Sensing Signal (Lee and Zhang 2015), which altogether control the expression of pyocyanin (the typical blue–green pigment interfering with host immune function and ciliary activity), exotoxins, proteases, and biofilm formation were discovered in *P. aeruginosa*. By modulating the expression of these virulence factors in response to population density, *P. aeruginosa* can adapt to its environment, shifting from acute infection to chronic colonization. Other important enzymes include elastases, which degrade host tissue components such as elastin and collagen, thereby facilitating tissue destruction and bacterial spread (Cigana et al. 2021). Moreover, the lipopolysaccharide (LPS) in the outer membrane can trigger a strong inflammatory response (Huszczynski et al. 2020)

Finally, *P. aeruginosa* is able to shift to an antibiotic‐resistant phenotype through both intrinsic mechanisms, such as efflux pumps, reduced permeability, and acquired resistance via horizontal gene transfer (Elfadadny et al. 2024). These traits significantly complicate treatment, making *P. aeruginosa* infections difficult to manage, especially in nosocomial settings.

On the other hand, the presence of this species in water, soil, plants, animals, and humans underscores the urgent need for an integrated surveillance and interdisciplinary approach within the One Health framework to mitigate its spread and clinical impact.

### 1.2 Importance of Alternative Antimicrobial Agents

Three levels of priority for the development of new antibiotics have been established by the World Health Organization (WHO): critical, high, and medium. The critical level includes several pathogens, including multiresistant, ESKAPE (*Enterococcus faecium*, *Staphylococcus aureus*, *Klebsiella pneumoniae*, *Acinetobacter baumannii*, *P. aeruginosa*, and *Enterobacter* spp.) (Mulani et al. 2019). Among them, *P. aeruginosa* is highly adaptable and, due to antibiotic overuse, has developed multidrug resistance (MDR) (Coșeriu, Mare, et al. 2023). The prevalence of *P. aeruginosa* MDR worldwide is around 25%–50%. In 2019, the ECDC reported that 3.4% of *P. aeruginosa* exhibited high‐level resistance to five antimicrobial classes (cephalosporins, penicillins, carbapenems, fluoroquinolones, and most phenicols). Numerous efforts are being made to develop and reduce the phenomenon. The incidence of multidrug and pan‐drug‐resistant strains is continuously increasing. For this reason, new strategies have been sought. One of these strategies is based on the combined use of antibiotic–essential oil (EO) conjugates (Roman et al. 2023).

EOs result from a preprocessing stage in which plants are subjected to various agronomic factors, such as harvesting, which can yield significantly different results. The high efficacy of EOs is attributed to their main active components, which contribute significantly to their antimicrobial activity. In general, these oils can interfere with various bacterial cellular processes, damaging membranes, altering ionic balance, and impairing the vital functions of the microbial cell. Indeed, the combination of antibiotics and EOs against *P. aeruginosa* has shown a significant increase in minimum inhibitory concentration (MIC) and biofilm inhibition. Therefore, their use in combination with specific antibiotics appears to be highly effective in minimizing drug doses and, consequently, reducing the risk of selecting for resistant bacteria (Sena et al. 2024).

**Figure 1:**
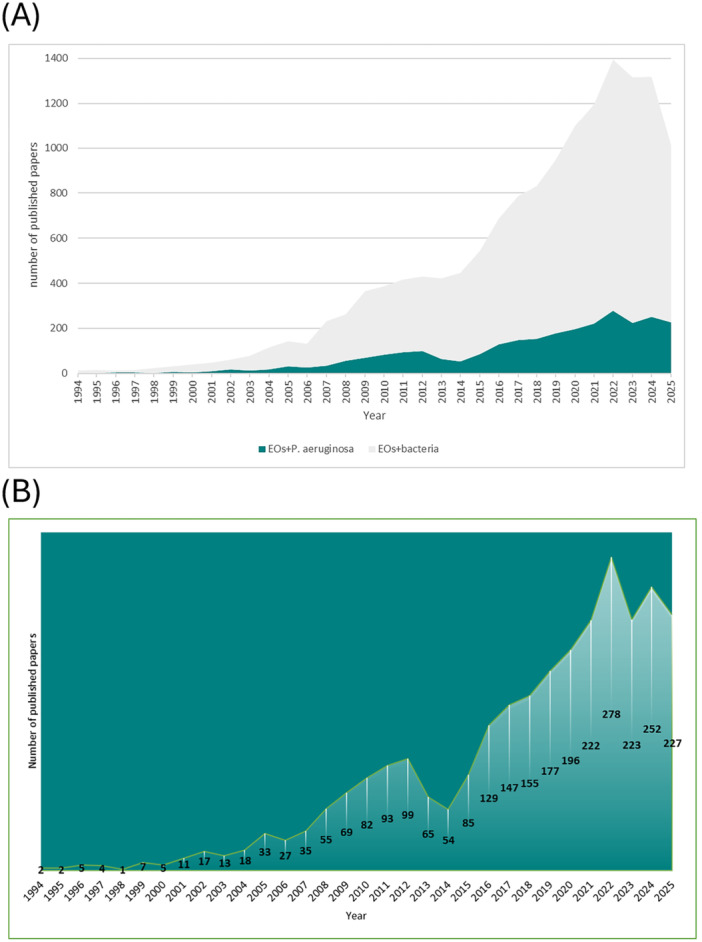
Number of published papers since 1994 according to ISI Web of Science (accessed in October 2025) by using “Essential oils AND bacteria” (gray area) or “Essential oils AND *P. aeruginosa*” (green–blue area) (A); a focus on the papers on “Essential oils AND *P. aeruginosa*”: The number of publications per year is reported in (B). EO, essential oil.

Since the early 1990s, scientific interest in the effects of EOs on bacteria, including *P. aeruginosa,* has steadily increased, mirroring the global rise in concern over antimicrobial resistance. A fast search on the Web of Science database by crossing “bacteria AND essential oils” (topic parameter) resulted in 12,066 works: while the number of published papers from 1994 to 2003 was in the tens, from 2004 to 2020 it was in the hundreds, and from 2021 to the present it has reached the thousands (Figure 1A). In parallel, by crossing “*P. aeruginosa* AND essential oils” we obtained 2778 publications since 1994. During the 1990s and early 2000s, only a limited number of studies on this topic were published, and moderate growth occurred between 2006 and 2012. A substantial increase in the number of published papers occurred from 2014 to the present (Figure 1B), and we hypothesize that this is related to increased awareness of the spread of antibiotic resistance and the development of more sophisticated approaches to studying *P. aeruginosa* virulence and biology. Besides the variation in the number of publications, a clear shift in the focus of the papers is evident. While early studies were mostly focused on the general antibacterial activity of EOs, the most recent ones mainly investigate the inhibitory effects of EOs on biofilm formation, QS, and the expression of virulence factors, without losing sight of synergistic interactions between EOs and conventional antibiotics. Moreover, other applications of EOs, such as food preservation, environmental management, and the design of antibiofilm devices, are increasingly emerging, thus opening the potential use of EOs against P. aeruginosa in other biotechnological contexts.

## 2 Chemical Composition of EOs

EOs are complex mixtures of small organic molecules extracted from plant materials such as flowers, leaves, and roots, typically through steam distillation. In this process, the volatile components are vaporized, and the resulting vapor is condensed into a liquid, allowing the organic phase (oil) to be separated from water (hydrosol) (Bakkali et al. 2008).

When the organic molecules in the EOs exhibit low (thermo)stability, alternative, and milder methods—such as cold pressing, solvent extraction, or advanced techniques like CO_2_ extraction—can be employed to capture these compounds, depending on their physical and chemical properties (Chemat et al. 2012). The main constituents of EOs are terpenes and their oxygenated derivatives, including phenols, alcohols, aldehydes, ketones, and esters (Figure 1). These volatile and lipophilic compounds are primarily responsible for the characteristic aroma and biological activities of EOs (Dhifi et al. 2016).

**Figure 2:**
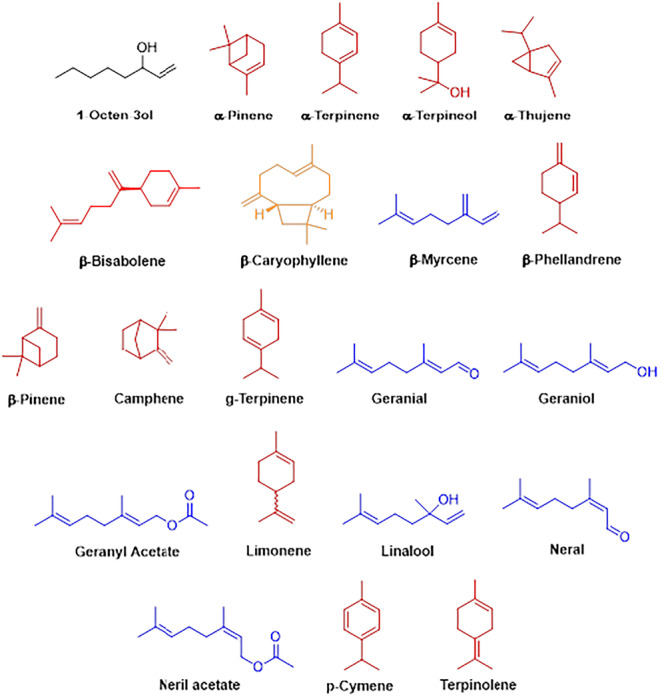
Chemical structure of the main components of essential oils. Linear and cyclic terpenes are marked in blue and red, respectively.

The molecular structures of these terpene derivatives are highly diverse and can be classified based on their structures. Linear terpenes (blue compounds in Figure 2), such as geraniol, geranial, neral, linalool, and β‐myrcene, often have lower boiling points and greater flexibility compared with cyclic terpenes (red compounds in Figure 2). This contributes to their characteristic aroma and their affinity for cell membranes. In contrast, cyclic terpenes—such as α‐ and β‐pinene, α‐ and *g*‐terpinene, α‐terpineol, α‐thujene, β‐phellandrene, camphene, limonene, and terpinolene—are more rigid and sterically hindered due to their six‐membered rings or bicycle structures, which reduces their interaction with linear lipids in cell membranes. More sterically hindered terpene derivatives, such as β‐caryophyllene (orange compound in Figure 2), are also found in several EOs, alongside simpler compounds like 1‐octen‐3‐ol (black compound in Figure 2).

### 2.1 Major Bioactive Compounds

**Figure 3:**
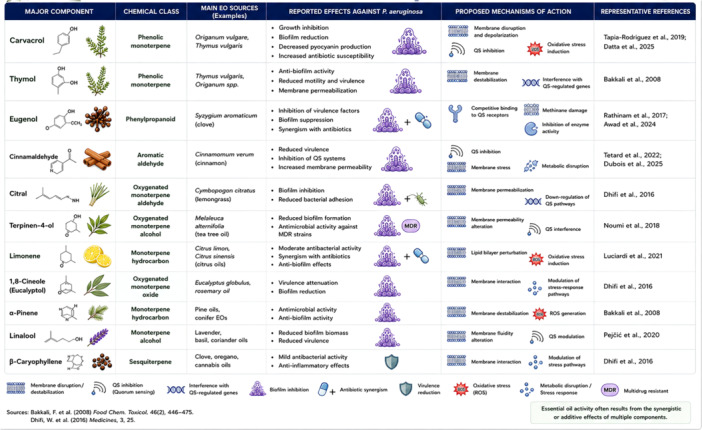
Major essential oil (EO) constituents with reported activity against *Pseudomonas aeruginosa*. The table summarizes representative bioactive components of EOs, their chemical classes, botanical sources, reported antibacterial and antivirulence effects against *P. aeruginosa*, and proposed mechanisms of action. Documented effects include growth inhibition, biofilm suppression, reduced motility and virulence‐factor production, enhanced antibiotic susceptibility, and activity against multidrug‐resistant strains. Proposed mechanisms involve membrane disruption or depolarization, increased membrane permeability, quorum‐sensing interference, oxidative‐stress induction, metabolic disruption, and modulation of stress‐response pathways. MDR, multidrug resistant; QS, quorum sensing; ROS, reactive oxygen species.

The concept of bioactive compounds is relatively broad and can be ambiguous in absolute terms. The bioactivity of a drug is typically associated with a specific therapeutic response or effects on a patient, such as antimicrobial, anti‐inflammatory, or anticancer activity. It is not uncommon for medical treatments to require more than a single compound and, in more complex cases, a mixture of compounds that work synergistically (Williamson 2001). This aspect is often underestimated in scientific studies, although it may play a primary role in understanding the rationale and mechanism beyond current medical treatments. It is also worth noting that the large number of compounds normally found in EOs, along with their possible interactions and synergistic effects, makes this type of study particularly challenging (Bakkali et al. 2008). In general terms, terpene derivatives and terpenoids excel in various areas, potentially enabling multitarget treatments due to their broad‐spectrum effects. Specifically, monoterpenes such as myrcene, limonene, linalool, carvacrol, and geraniol belong to this family of broad‐spectrum terpenoids and demonstrate a wide range of applications. Similarly, sequiterpenes also exhibit broad bioactivity, although most of them—with the exception of the β‐caryophyllene—are not commonly found in EOs (Bakkali et al. 2008; Figueiredo et al. 2008). The biological activity of EO constituents is not uniformly distributed across chemical classes (Figure 3). Phenolic monoterpenes such as carvacrol and thymol are generally associated with strong antimicrobial activity because their hydroxyl groups increase their ability to interact with and disrupt bacterial membranes. In contrast, hydrocarbon monoterpenes, such as limonene or myrcene, often exhibit weaker direct antibacterial effects, but may contribute to QS inhibition, membrane permeabilization, or synergistic interactions with other EO components. Oxygenated monoterpenes, including linalool and geraniol, occupy an intermediate position and frequently display both antimicrobial and anti‐inflammatory properties. This diversity of activities supports the view that EO efficacy frequently results from the combined action of multiple constituents rather than from a single dominant compound. Such synergistic interactions may explain why whole EOs often display greater biological activity than isolated molecules despite the lower concentration of individual active components.

### 2.2 Variability in Composition Based on Plant Species and Extraction Methods

A major challenge in evaluating and comparing the biological activities of EOs is their inherent variability in chemical composition, which is influenced by multiple botanical, environmental, and technical factors. In fact, both the qualitative and quantitative profiles of an EO can differ substantially among plant species, and even within the same plant species according to the subspecies. Very recently, Dodoš et al. (2025) related the genetic diversity of natural populations of two *Satureja subspicata* subspecies (*S. subspicata* ssp. *subspicata* and *S. subspicata* ssp. *liburnica* Silic.) by using the composition of their EOs as a variable. A total of 133 molecules, mainly belonging to sesquiterpenes and monoterpenes, have been identified in the EO of *S. subspicata* subsp. *liburnica* and 132 in the EO of *S. subspicata* subsp. *subspicata*. Compounds such as α‐pinene, caryophyllene oxide, γ‐muurolene, and viridiflorol characterized geographically distant populations, while higher levels of caryophyllene oxide distinguished ssp. *liburnica* from ssp. *subspicata*. Another variable is the organ used (leaves, flowers, seeds, resins, or roots) to extract EOs (Zawadzińska et al. 2025; Rawat et al. 2025).

In addition, environmental variables—such as soil characteristics, climate, altitude, and phenological stage—further contribute to fluctuations in metabolite production and in their biological qualities. The variability in EO composition according to climate was assessed in the seed extract of *Bunium persicum* Bioss growing in seven zones of the Northwestern‐Himalayan region with different altitudes ranging from 1587 to 3722 m (M. H. Khan, Dar, et al. 2023). Molecules such as *p*‐cymene, γ‐terpinene, d‐limonene, cumic aldehyde, and 1,4‐*p*‐menthadien‐7‐al showed high quantitative variability according to the geographical zone and climate. These differences in the EO chemical composition and oil content were related not only to different climate zones and altitudes, but also to the plant genetic variability observed in the populations.

Moreover, time of harvest, phenological phases (viz., reproductive or vegetative), soil, and climatic conditions can affect the EOs composition. In their work published in 2022, Rathore and Kumar (2022) characterized the variability in EO content extracted from *Rosmarinus officinalis* L., growing in the Himalayan region, according to the harvest seasons. The highest EO amount was obtained from plants sampled in autumn, while the lowest was from rosemary harvested in summer. The main components of the rosemary EO were 1,8‐cineole, α‐pinene, camphor, camphene, β‐pinene, and terpinen‐4‐ol with some minor compounds (area < 3%) as β‐myrcene, *cis*‐sabinene hydrate, α‐terpinene, borneol, linalool, α‐terpineol, α‐phellandrene, and β‐caryophyllene. However, higher levels of 1,8‐cineole, β‐pinene, *cis*‐sabinene hydrate, and terpinen‐4‐ol were detected in EO extracted from plants harvested in the rainy season. β‐Caryophyllene concentration was higher in the rainy season compared with summer and disappeared in the EO extracted from rosemary harvested in autumn, while a higher amount of α‐pinene was recorded in the summer season compared with autumn and rainy season. The different compositions recorded according to the harvest time resulted in diverse biological activity against opportunistic human pathogens, with the EO extracted from plants harvested during the rainy season being the most active against *S. aureus*.

Equally important are the extraction methods employed: steam distillation, hydrodistillation, cold pressing, and solvent‐ or CO_2_‐based extractions can produce oils with markedly different concentrations of monoterpenes, sesquiterpenes, phenylpropanoids, and other volatile compounds. These differences influence not only aroma and physicochemical properties, but also antimicrobial potency, anti‐virulence activity, and cytotoxic profiles. As an example, the efficacy of different drying methods on the amount and quality of *Thymus daenensis* EO was assessed by Dehghani et al. (2018): in this work, the authors considered as first variable the pre‐drying under sun and as second variable the drying methods (sun drying, oven drying at different temperatures ranging from 35°C to 55°C, shade drying, vacuum drying, and microwave drying) as the second factor. The EO content was significantly impacted by the combination of pre‐drying with the different drying procedures. In detail, the highest EO contents were obtained by oven‐drying and vacuum oven‐drying at 35°C without a pre‐drying treatment. The greatest content of thymol was acquired in the vacuum oven‐drying at 55°C, but the other detectable EO components were reduced.

Consequently, variability in EO composition complicates reproducibility, limits cross‐study comparability, and underscores the need for rigorous chemical characterization, especially when assessing their biological effects.

## 3 Mechanisms of Action of EOs Against *P. aeruginosa*

**Figure 4:**
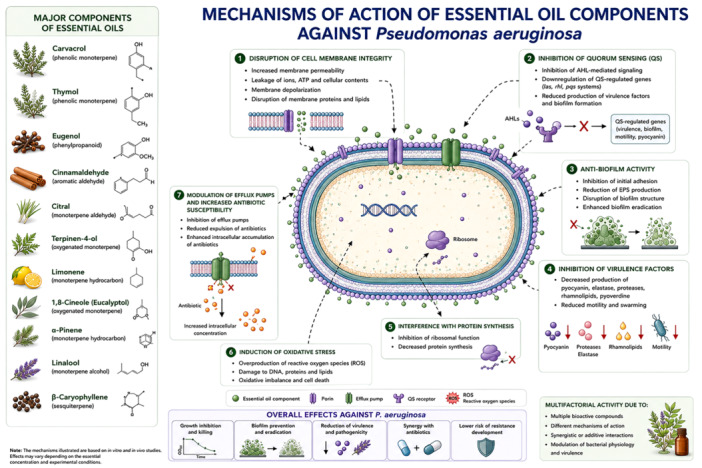
Proposed mechanisms of action of major essential oil (EO) components against *Pseudomonas aeruginosa*. The figure summarizes the multifactorial antibacterial and anti‐virulence activities exerted by major EO constituents, including phenolic monoterpenes (carvacrol and thymol), phenylpropanoids (eugenol), aldehydes (cinnamaldehyde and citral), and terpene‐rich compounds (limonene, terpinen‐4‐ol, 1,8‐cineole, linalool, and α‐pinene). These bioactive molecules can affect *P. aeruginosa* through several complementary mechanisms: (1) disruption of cell membrane integrity, leading to increased permeability, ion leakage, membrane depolarization, and loss of cellular homeostasis; (2) inhibition of quorum‐sensing (QS) signaling pathways through interference with acylated homoserine lactone (AHL)‐mediated communication and downregulation of QS‐regulated genes; (3) inhibition of biofilm formation and maturation; (4) attenuation of virulence‐factor production, including pyocyanin, proteases, elastase, rhamnolipids, and bacterial motility; (5) interference with protein synthesis and ribosomal activity; (6) induction of oxidative stress through reactive oxygen species (ROS) accumulation; and (7) modulation of efflux pump activity, resulting in enhanced intracellular accumulation of antibiotics and increased bacterial susceptibility. Collectively, these effects contribute to growth inhibition, biofilm disruption, reduced pathogenicity, and potentiation of conventional antibiotics, highlighting the potential of EOs as multifunctional antimicrobial and anti‐virulence agents against *P. aeruginosa*.

EOs exert a multifaceted inhibitory action against P. aeruginosa, targeting both bacterial viability and key virulence pathways. Their complex mixtures of monoterpenes, phenolics, and aldehydes can disrupt membrane integrity, increase permeability, and interfere with bacterial motility, ultimately impairing bacterial growth and environmental fitness. Beyond these direct antibacterial effects, EOs also modulate several regulatory systems essential for *P. aeruginosa* pathogenicity and survival, including QS, biofilm development, antibiotic resistance, and the synthesis of virulence factors, such as pyocyanin, elastase, and rhamnolipids. The multifaceted nature of EO activity against *P. aeruginosa*, highlighting how different chemical classes target distinct but interconnected bacterial processes, is illustrated in Figure 4. Phenolic compounds are primarily associated with membrane disruption and increased permeability, whereas aldehydes such as cinnamaldehyde have been linked to both membrane damage and interference with QS regulation. Monoterpene alcohols, including geraniol and linalool, appear particularly effective in modulating virulence‐related pathways and biofilm development. These observations suggest the existence of structure–activity relationships in which specific functional groups, such as hydroxyl or aldehyde moieties, influence the ability of EO constituents to interact with bacterial membranes or regulatory proteins. The simultaneous targeting of multiple pathways may explain both the broad‐spectrum activity of EOs and their capacity to enhance antibiotic efficacy, while reducing the likelihood of resistance development. The main mechanisms of action of EOs against *P. aeruginosa* are detailed below.

### 3.1 Disruption of Cell Membranes

The main antimicrobial mechanism described in the literature by which EOs act against *P. aeruginosa* is the disruption of bacterial cell membrane integrity. Due to their marked lipophilicity, many EO constituents, including phenolic compounds such as carvacrol and thymol, monoterpenes such as geraniol and limonene, and aldehydes such as cinnamaldehyde, can partition into the lipid bilayer of *P. aeruginosa*, altering the highly ordered structure of the outer membrane and the cytoplasmic membrane (Helander et al. 1998). In *P. aeruginosa* the outer membrane represents a major permeability barrier because of its dense LPS layer and low fluidity, but the EO component can destabilize this arrangement by increasing membrane fluidity, disrupting LPS packing, and enhancing permeability (Helander et al. 1998; Haripriyan et al. 2025). This increase in membrane fluidity can also facilitate the entry of other antimicrobial compounds, potentially enhancing synergistic effects. Once the outer membrane is compromised, these compounds reach the cytoplasmic membrane, where they induce loss of membrane potential, dissipation of the proton motive force, leakage of intracellular ions and metabolites, and a general collapse of cellular homeostasis (Ultee et al. 2002). Experimental data, such as increased uptake of propidium iodide, release of nucleic acids and proteins, and changes in electrical conductivity, confirm the membrane‐damaging activity of EOs in *P. aeruginosa* (Nazzaro et al. 2013). The specific effects on membrane integrity also depend on the chemical class of the EO constituents, as phenols, monoterpenes, and aldehydes interact differently with lipid bilayers. Perhaps, the disruption of membrane organization can also interfere with systems that maintain lipid asymmetry in the outer membrane, including the MLA (Maintenance of Lipid Asymmetry) pathway, making the bacterium more susceptible to environmental stress and antimicrobial compounds (Munguia et al. 2017). Indeed, by compromising the structural and functional integrity of the bacterial envelope, EOs reduce viability and increase the susceptibility of *P. aeruginosa* to antibiotics, supporting their potential use as antimicrobial or adjuvant agents against infections involving highly resistant strains (Novelli et al. 2025).

### 3.2 Effects on QS

**Figure 5:**
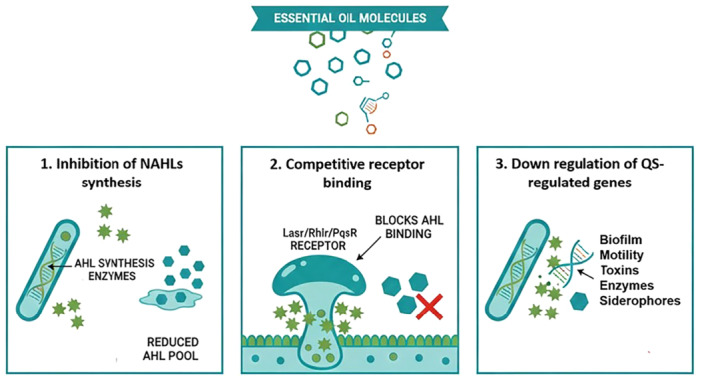
Overview of the mechanisms by which essential oils (EOs) or their pure compounds interfere with bacterial quorum sensing (QS): (1) inhibition of autoinducer synthesis: EOs molecules disrupt the activity of AHL‐synthesizing enzymes, resulting in a reduced pool of QS autoinducers; (2) competitive receptor binding: EOs constituents competitively interact with QS receptors (e.g., LasR, RhlR, and PqsR), preventing the binding of native AHL signals and thereby blocking downstream signal transduction; (3) downregulation of QS‐regulated genes: by impairing signal synthesis or receptor activation, EOs lead to decreased expression of key quorum‐regulated traits, including biofilm formation, motility, toxin production, extracellular enzymes, and siderophore biosynthesis. AHL, acylated homoserine lactone; NAHLs, *N*‐acyl homoserine lactones.

One of the most intriguing mechanisms by which EOs exert anti‐virulence activity against *P. aeruginosa* is their ability to inhibit QS, the cell–cell communication system that controls key pathogenic traits in response to bacterial density. Many EOs and their major constituents—especially phenolic (e.g., carvacrol and thymol), aldehydic (e.g., citral), and terpene‐rich molecules—can disrupt QS signaling without necessarily exerting strong bactericidal pressure. This interference may occur through several complementary mechanisms, including:

i. inhibition of signal molecules (*N*‐acyl homoserine lactones, NAHLs) synthesis, thus reducing the pool of autoinducers available to trigger QS cascades (Haripriyan et al. 2025; Deryabin et al. 2019; Tapia‐Rodriguez et al. 2019);
ii. competitive binding to QS receptors (LasR, RhlR, and PqsR), thus preventing activation of downstream gene expression (Datta et al. 2025; Rathinam et al. 2017);
iii. downregulation of QS‐regulated genes (Qaralleh et al. 2024; Al‐Helo et al. 2025) (Figure 5).

It should be specified that most mechanistic studies investigating the inhibition of QS in *P. aeruginosa* via competitive binding to LasR, RhlR, and PqsR receptors have focused on individual compounds derived from EOs, rather than on whole EOs themselves. These compounds can directly interact with the ligand‐binding pockets of QS receptors. The preference for testing single compounds arises from the chemical complexity and variability of EOs. Moreover, the specificity of QS receptors makes it difficult to attribute competitive binding effects to the oil as a whole, whereas single compounds allow precise modeling of receptor interactions.

By targeting communication rather than viability, EOs reduce the selective pressure for resistance and can attenuate crucial virulence behaviors such as biofilm maturation, motility, and toxin production. These properties make EOs particularly promising as anti‐virulence agents and as candidates for combination strategies to manage *P. aeruginosa* infections, especially those characterized by chronic biofilms or persistent inflammation.

### 3.3 Inhibition of Biofilm Formation

EOs exhibited a pronounced inhibitory effect on the early stages of biofilm formation in *P. aeruginosa*, whose persistence in clinical settings is strongly associated with its ability to establish structured, multidrug‐tolerant biofilms. Treatment with subinhibitory concentrations of selected EOs significantly reduced initial bacterial adhesion and impaired the development of organized biofilm architecture, as demonstrated by decreased crystal‐violet biomass quantification and disrupted microcolony maturation. These findings are reported in different studies indicating that EOs activity against *P. aeruginosa* biofilms is primarily mediated through anti‐virulence and anti‐adhesive mechanisms rather than direct bactericidal effects (Pejčić et al. 2020; Lu et al. 2022; Benaissa et al. 2024).

At the molecular level, phenolic EO constituents such as thymol, eugenol, carvacrol, and cinnamaldehyde have been shown to interfere with QS regulatory networks, including the las, rhl, and pqs systems, resulting in reduced expression of QS‐controlled virulence determinants and extracellular polymeric substance biosynthesis (Benaissa et al. 2024; Alibi et al. 2020; Awad et al. 2024). Sub‐MIC exposure to clove, oregano, thyme, basil, and cinnamon EOs has been associated with the downregulation of QS‐related genes such as lasI, lasR, pel, and aprA, together with a marked reduction in motility, pyocyanin production, and early biofilm stabilization, both in reference strains and in clinical multidrug‐resistant isolates (Pejčić et al. 2020; Awad et al. 2024; Brożyna et al. 2025). In parallel, EOs exert biophysical effects on bacterial cells, including increased membrane permeability and leakage of intracellular components, leading to impaired metabolic activity required for irreversible surface attachment and biofilm maturation (Lu et al. 2022; Almanaa et al. 2021). The observed downregulation of genes involved in flagellar motility and pili‐mediated adhesion (e.g., fliC and pilA) further supports the notion that EOs primarily target the transition from reversible adhesion to structured biofilm formation (Pejčić et al. 2020; Alibi et al. 2020). Importantly, these effects occur at concentrations that do not substantially affect planktonic viability, reinforcing the concept of EOs as anti‐biofilm and anti‐virulence agents rather than conventional antibiotics.

Recent advances in EO delivery strategies further support this paradigm. Nanoemulsions, hydrogels, and nanostructured lipid or archaeolipid carriers have been shown to enhance EO stability, control release kinetics, reduce cytotoxicity, and potentiate antibiofilm efficacy, particularly when EOs are combined with conventional antibiotics, such as levofloxacin or tobramycin (Razdan et al. 2023; Perez et al. 2022; Marzban et al. 2025). These combinatorial approaches improve biofilm penetration and restore antibiotic susceptibility by weakening the biofilm matrix and disrupting QS‐regulated defense mechanisms.

Collectively, these findings indicate that EOs attenuate *P. aeruginosa* pathogenicity by preventing biofilm establishment and early maturation rather than exerting strong bactericidal pressure on mature biofilms. In the context of the increasing prevalence of multidrug‐resistant *P. aeruginosa*, EO‐based adjuvant strategies may represent a valuable complementary approach to conventional antimicrobial therapy. However, recent strain‐resolved analyses have revealed substantial interstrain variability in EO susceptibility, highlighting the need for standardized evaluation frameworks and an EO stewardship approach to ensure reproducibility and clinically meaningful translation (Brożyna et al. 2025). Further mechanistic studies and in vivo validation are warranted to define optimal EO compositions, delivery systems, and combinatorial regimens with established antimicrobials.

## 4 Synergistic Effects With Antibiotics

Treating bacterial infections is a major challenge for modern medicine due to the spread of antibiotic resistance. Medical research focuses on developing new inhibitory molecules against MDR bacteria; however, designing new classes of antibiotics is not always easy. It should be noted that the new antibiotics that entered the market in recent decades are derived from pre‐existing antibiotics, with chemical variations. This lack of progress in the research for new antibiotic compounds reveals the need for innovative strategies to treat bacterial infections. One of these is the combination of conventional antibiotics with plant‐derived molecules with antimicrobial properties, such as various plant extracts, phytochemicals, and EOs (Roman et al. 2023; Neagu et al. 2024). The diversity of the components of the EOs confers a wide variety of biological features, some of which represent valid alternatives or supplements to synthetic compounds (Bekka‐Hadji et al. 2022). This approach seems to be effective in overcoming the mechanisms at the base of bacterial resistance by (i) increasing the susceptibility of bacterial pathogens to antibiotics through the enhancement of the drug's effectiveness or (ii) producing a more intense antimicrobial effect. In fact, certain components of EOs can suppress the activity of bacterial efflux pumps, which can expel antibiotics from cells (Abbad et al. 2025). Moreover, EOs or their major constituents can enhance the activity of antibiotics against MDR bacteria, including *P. aeruginosa*, by reducing MICs and restoring drug susceptibility that would otherwise be lost. For instance, only the combination of Cinnamaldehyde (the main component of cinnamon EO) and ciprofloxacin showed a synergistic effect against *P. aeruginosa,* whereas the other 16 of 24 EO/antibiotic combinations showed additive effects (Simbu et al. 2024). On the contrary, only a limited effect of *Citrus bergamia* EO coupled with tetracycline was observed in *P. aeruginosa*, where the MIC for the combination EO + antibiotic was reduced fourfold (Sena et al. 2024).

Overall, recent experimental data on *P. aeruginosa* remain relatively scarce compared with those for other bacteria (e.g., *Escherichia coli*); however, studies on purified EO components frequently report synergistic interactions with antibiotics, which support a general potentiation mechanism. Moreover, most evidence currently derives from in vitro studies, and further investigations are required to validate these synergistic effects in vivo and to assess their safety and clinical applicability.

## 5 In Vitro Studies on Antimicrobial Activity

### 5.1 Methods for Testing Antimicrobial Activity

While methods for assessing the efficacy of antibiotics are standardized and guidelines have been established by the Clinical and Laboratory Standards Institute (CLSI) and European Committee on Antimicrobial Susceptibility Testing (EUCAST) since 1997, no regulatory procedures have been established to measure the antimicrobial properties of EOs. As a consequence, scientists working on EO‐mediated antibiosis often adapt methods used for conventional antibiotics, which are not always adequate for EOs, as they are composed of both volatile and lipophilic molecules. Among these approaches (for an excellent and recent review, see Hulankova 2024), MIC determination, particularly when performed in 96‐well microplates, is the most commonly used method to assess bacterial susceptibility to EOs. In addition to microdilution assays, other techniques, such as agar dilution, disk diffusion, and broth dilution, have been employed, each with specific advantages and limitations when dealing with volatile compounds. Being the culture medium recommended by EUCAST and CLSI, Mueller–Hinton is the most frequently used by scientists working on EOs, but it is not suitable for the growth of mildly fastidious microorganisms. Therefore, using different culture media such as Tryptic Soy Broth, or Luria–Bertani broth, or Nutrient broth leads to variability among results. Moreover, experimental outcomes may vary according to the solvents used to dilute EOs. While dimethyl sulfoxide is most commonly employed, ethanol and methanol are also used as alternative solvents (Hulankova 2024). The choice of solvent can significantly affect both the solubility of EO components and their antimicrobial performance. When interpreting data from these assays, it should be noted that vapor transfer between wells can influence results, particularly when volatile fractions are involved. Furthermore, optical density measurements may be performed at different wavelengths (typically ranging from 600 to 650 nm), depending on the spectrophotometric parameters chosen for bacterial growth assessment. Last but not least, a general consensus on the units used to express MIC values is lacking, further complicating comparisons of results across studies (Hulankova 2024). The lack of standardized protocols hampers the reproducibility and comparability of data among studies, thus limiting the development of EO‐based antimicrobial formulations. Therefore, the establishment of harmonized testing protocols specifically designed for EOs would be crucial to ensure consistent, reproducible, and reliable evaluation of their antimicrobial potential.

### 5.2 Summary of Studies Showing Efficacy: Comparison of EOs From Different Plants

The literature summarized in Table 1 provides an overview of EOs used as alternative or adjunctive strategies against *P. aeruginosa*.

**Table 1:** A selection of the literature on the use of essential oils (EOs) against *Pseudomonas aeruginosa*.

Essential oil (plant species)	Main results	Reference
*Mentha piperita* (peppermint)	In *P. aeruginosa* PAO1, peppermint EO inhibited quorum‐sensing (QS)–regulated virulence without affecting bacterial growth. At sub‐MIC concentration (6.4% v/v), the EO at a concentration of 3% reduced elastase, protease, and chitinase activities by up to 80%, 76%, and 78%, respectively. Pyocyan and exopolysaccharide production decreased by 85.2% and 76%, respectively. Moreover, swarming motility was suppressed by 81.3%. Molecular docking analysis showed that menthol binds to the LasR receptor, competes with AHL signal, and disrupts *las* and *pqs* QS systems.	Husain et al. (2015)
*Citrus reticulata* (mandarin)	Mandarin EO is composed of ɑ‐pinene, γ‐terpinene, and myrcene. Although it did not inhibit bacterial growth at 4 mg/mL, it suppressed biofilm formation by up to 82% in the model strain *P. aeruginosa* ATCC 27853 and by up to 73% in *P. aeruginosa* HT5 isolated from a patient with food poisoning. The EO at a concentration of 4 mg/mL also reduced biofilm cell viability by 33% and 59% of the ATCC and HT5 strains, respectively. These effects are associated with a decrease in AHL production (49% for the ATCC strain and 38% for the HT5 strain) and a 77% reduction in elastase activity.	Luciardi et al. (2016)
*Calamintha nepeta* Savi subsp. *glandulosa* (Req.) Ball (lesser calamint), *Foeniculum vulgare* (fennel), *Ridolfia segetum* (false fennel)	The ability of 89 EO samples, obtained from *C. nepeta* Savi subsp. *glandulosa* (Req.) Ball, *F. vulgare*, and *R. segetum* to inhibit *P. aeruginosa* was evaluated. Main constituents of EOs were estragole, phellandral, d‐limonene, pulegone, and chrysanthenone. The concentration range of 25–0.045 mg/mL completely inhibited bacterial growth. The biofilm formation inhibition was detected from a concentration of 25 mg/mL, with a specific effect according to the EO characteristics and composition (inhibition was considered if the reduction in biofilm was greater than 50% and 46%, and the concentrations of EOs were 48.8 g/mL and 3.125 mg/mL, respectively). Almost all fennel and false fennel EOs samples inhibited the growth of *P. aeruginosa* biofilm, with a dose‐dependent effect. Except for one sample, lesser calamint samples allowed biofilm development. RS extracts inhibited biofilm growth.	Artini et al. (2018)
*Lavanda sumian* (lavandin), *Lavanda grosso* (hybrid lavanda)	*L. sumian* EO showed lower antimicrobial activity compared with *L. grosso* EO against 16 multidrug‐resistant *P. aeruginosa* strains. *L. sumian* EO was ineffective on 11 *P. aeruginosa* strains at the concentration of 16%, while *L. grosso* EO was effective on the same number of *P. aeruginosa* at the concentration of 8%. Both lavender EOs tested did not show cytotoxic effect at very low concentrations, on human Wong–Kilbourne derivative conjunctiva cells. The EOs extracted from *L. sumian* and *L. grosso* significantly reduced nitric oxide synthase (NOS) activity on murine macrophage (J774.1A).	Donadu et al. (2018)
*Melaleuca alternifolia* (tea tree)	EO extracted from *M. alternifolia*, mainly composed of terpinen‐4‐ol, γ‐terpinene, and α‐terpinen, was tested for its efficacy against *P. aeruginosa* PAO1 focusing on swarming and pyocyanin production.	Noumi et al. (2018)
	The three considered concentrations of EO (50, 75, and 100 µg/mL) inhibited both the swarming and the pyocyanin synthesis. The highest swarming suppression (33.33%) was achieved with EO at 100 µg/mL.	
*Salvia officinalis* (common sage)	The EO of *S. officinalis* is composed primarily of *cis*‐thujone, camphor, *trans*‐thujone, and 1,8‐cineole. Those compounds demonstrated significant antibacterial activity against *P. aeruginosa* in a murine wound‐infection model. The EO exhibited an MIC of 0.125 mg/mL and effectively reduced bacterial density in tissues while accelerating re‐epithelialization and granulation. Treatment with 4% sage EO reduced pro‐inflammatory cytokines like IL‐6, TNF‐α, IL‐1β, and increased the expression of FGF‐2, VEGF, Bcl‐2, and cyclin‐D1, indicating an antimicrobial and tissue‐repair‐promoting effect. Those findings suggest that sage EO not only counters *P. aeruginosa* infection, but also modulates inflammation and enhances wound healing.	Farahpour et al. (2020)
*Satureja khuzistanica* (marzeh khuzistani)	*P. aeruginosa* can resist antibiotics thanks to efflux pumps such as MexEF–OprN and MexXY–OprM. The efficacy of the EO extracted from *S. khuzistanica* on the efflux pump activity of six strains of *P. aeruginosa* isolated from the pus and wound specimens of burn patients in Tehran was tested. MICs were determined for gentamicin and norfloxacin as positive controls. To measure differential expression of efflux pump genes, reverse transcription‐polymerase chain reaction was used. The MIC values of *S. khuzistanica* EO ranged from 6 to 12 μg/mL, and the effect of the combination with norfloxacin and gentamicin increased up to eightfold. The expression of MexY and MexE was reduced by 5 and 9.7‐fold, respectively, after treatment with *S. khuzistanica* EO.	Iman Islamieh et al. (2020)
*Ocimum basilicum* (basil), *S. officinalis* (common sage)	Basil EO is rich in linalool, (*E*)‐anethole, and methyl chavicol, while sage EO is mainly composed of oxygenated monoterpenes with ɑ‐thujore, camphor, and eucalyptol. These compounds act by interfering with the synthesis of quorum‐sensing signaling molecules (*N*‐acyl‐homoserine lactones) that regulate the *las* and *rhl* systems. The basil and sage EOs inhibited biofilm formation (up to 99% and 99.6%, respectively) and reduced mature biofilm by 74.7%–99.7% and by 81.3%–98.3%, respectively. They also decreased swimming, swarming, and twitching motility and suppressed pyocyanin production (basil, by 13.3%–55.6%; sage, by 58.8%). The antivirulence action is associated with disruption of QS‐mediated regulation, leading to reduced production of extracellular polymeric substances and diminished structural stability of the biofilm matrix.	Pejčić et al. (2020)
*Nerium oleander* (oleander)	*N. oleander* EO showed an antibiofilm activity against *P. aeruginosa* due to its richness in oleandrin, neriin, and oleandrigenin. This EO at 200 μg/mL inhibited biofilm formation and reduced bacterial viability. The EO binds to the bacterial surface, permeates inside the cell wall, and affects the integrity of the cytoplasmic membrane. The signaling factors lose their role, and bacteria stop producing exopolysaccharides.	Almanaa et al. (2021)
*Cinnamomum zeylanicum* (cinnamon), *Eugenia caryophyllus* (clove)	The EOs of *C. zeylanicum* (CLO) and *E. caryophyllus* (CO), both rich in eugenol, were incorporated into chitosan/polyvinyl alcohol (CS/PVA) films (30/70, w/w) to enhance their antimicrobial performance and flexibility. Films loaded with 1% and 10% cinnamon showed significantly higher antimicrobial activity than unloaded films. The CS/PVA + CLO 10% formulation exhibited the strongest inhibition against *Staphylococcus aureus* and, consistently with the intrinsic resistance of Gram‐negative bacteria, a moderate effect against *P. aeruginosa*. The results suggest that eugenol‐rich EO incorporation enhances the antibacterial potential of CS‐based films, while maintaining desirable mechanical properties.	Antunes et al. (2021)
*Lavandula angustifolia* (lavender), *Rosmarinus officinalis* (rosemary), and *S. khuzistanica* (marzeh khuzistani)	The effect of five EO nanoemulsions (EO‐nano) obtained from *L. angustifolia, R. officinalis*, and *S. khuzistanica*, and two EO constituents (carvacrol and 1,8‐cineol), was studied on *P. aeruginosa* strain PAO1. EO‐nano improves the antibacterial activity of EOs between 4 and 8 times compared with bulk oils. Nanoformulation of *S. khuzistanica* showed the highest activity against *P. aeruginosa* biofilm, with 89.53% ± 4.88% inhibition and 56.65% ± 7.65% eradication effect.	Ghaderi et al. (2020)
*Citrus limon* (lemon)	*Citrus* EOs and their components, particularly lemon EO, lemon terpenes, and lemon essence, contain high amounts of monoterpene hydrocarbons, with limonene as the major component. All lemon oils significantly inhibited *P. aeruginosa* ATCC 27853 and HT5 biofilm formation in a dose‐dependent manner (20%–65% at 0.1–4 mg/mL), with lemon essence being the most effective. Limonene alone had limited activity, suggesting synergistic interactions among oil constituents. Lemon EO, lemon terpenes, and lemon essence reduced the production of *N*‐acyl homoserine lactones (NAHLs) and, subsequently, biofilm formation, the expression of virulence factors, and swarming motility.	Luciardi et al. (2021)
*R. officinalis* (rosemary) and antibiotic cefepime co‐encapsulated in multiple lipid nanoparticles	Rosemary EO and the antibiotic cefepime were co‐encapsulated in multiple lipid nanoparticles (MLNs). GC–MS analysis of rosemary EO identified 1,8‐cineole (23.8%), α‐pinene (16.2%), camphor (15.3%), and camphene (9.8%) as the main components. Microbiological assays against three *P. aeruginosa* strains, with different cefepime resistance profiles, showed that free EO possessed moderate antibacterial activity (MIC 40–80 mg/mL), while encapsulation within MLNs reduced the MIC by more than 100‐fold (0.6 mg/mL for *P. aeruginosa* ATCC 9027). Encapsulated and free cefepime showed similar efficacy, with the encapsulated one displaying greater thermal and temporal stability. Co‐encapsulation of EO and antibiotic proved technically feasible, demonstrating the ability to combine a lipophilic and a hydrophilic active compound within a single lipid matrix. Encapsulation in lipid nanoparticles represents an effective and stable delivery system capable of enhancing the antibacterial activity of EOs and improving the preservation of hydrophilic antibiotics, offering promising perspectives for therapeutic applications against multidrug‐resistant bacteria.	Ben‐Khalifa et al. (2021)
*Coridothymus capitatus* (conehead thyme)	*C. capitatus* EO showed inhibition of biofilm formation and reduction in mature biofilm, in a variable percentage (from 60% to 40%), on about half of the tested strains of *P. aeruginosa*. The treatment also induced a drastic reduction in pyocyanin production (between 84% and 100%), swarming and swimming abilities of almost all *P. aeruginosa* strains.	Vrenna et al. (2021)
*Thymus vulgaris* (thyme), *M. alternifolia* (tea tree), *O. basilicum* (Basil), *R. officinalis* (rosemary), *Eucalyptus* spp., *Mentha arvensis* (menthol mint), *Lavandula* spp. (lavender)	Since the topical treatment with pure EOs may cause allergic reactions and skin irritation. Therefore, EO volatile fractions represent a suitable alternative for the therapy of bacterial infections. The efficacy of thyme, tea tree, basil, rosemary, eucalyptus, menthol mint, and lavender, in both volatile and liquid phases, on *P. aeruginosa* growth, biofilm formation, and planktonic cell release was evaluated. Overall, liquid and volatile forms of rosemary EO and tea tree EO demonstrated significant antibiofilm effectiveness. The results suggested that these seven EOs could be used to treat *P. aeruginosa* infections.	Brożyna et al. (2022)
*Citrus paradisi* (grapefruit), *C. reticulata* (mandarin)	At low concentrations (0.125%), the EOs inhibited biofilm formation by 40%–50%, also reducing metabolic activity, autoinducer (NAHLs) production, and elastase activity, without affecting bacterial growth. The main component, limonene, showed a weaker effect compared with the complete EOs. The EOs protected the nematode *Caenorhabditis elegans* from paralysis and death caused by *P. aeruginosa*, PAO1 and PA14, with a recovery of 20%–40% of the animals. Limonene alone showed a lower protective effect (~ 8%). The data suggest that the EOs interfere with the bacterial QS system, reducing the expression of cyanidric acid.	D'Almeida et al. (2022)
*Origanum vulgare* (oregano)	The efficacy of oregano EO and blue light (BL) at 405 nm, alone or in combination, was tested against planktonic cells as well as mature biofilms of the multidrug‐resistant *P. aeruginosa* ATCC19660 and HS0065. The combination of the two treatments synergistically killed *P. aeruginosa* cells and decreased biofilms by 7 log10 units. The activity of EO + blue light was also assessed in 8‐week‐old BALB/C mice with acute burn wounds infected with *P. aeruginosa*, showing that the treatment eradicated acute or biofilm‐associated infection without any adverse effects on the skin.	Lu et al. (2022)
*T. vulgaris* (thyme)	Thyme EO extracted at the beginning and at the end of the flowering period inhibited *P. aeruginosa* LPS adhesion and showed synergistic effects when combined with its main constituent thymol. Measurements of the activity of the antioxidant enzymes (peroxidase, catalase, and superoxide dismutase), as well as the determination of the total antioxidant capacity of THP‐1 cells activated by *P. aeruginosa*, revealed that both thymol and thyme EO increased the catalase and superoxide dismutase activity, as well as the antioxidant capacity of THP‐1 cells. Measurements of the mRNA expression of pro‐inflammatory cytokines and the secreted protein levels in THP‐1 cells activated by the bacterial strain LPS showed that thyme EO extracted at the beginning of the flowering time suppressed the synthesis of IL‐6, IL‐8, IL‐1β, and TNF‐α.	Pandur et al. (2022)
*T. vulgaris* (thyme)	Co‐delivery of *T. vulgaris* EO and tobramicyn by nanostructure archeolipid carriers (NACs) can support nebulization as well as improve antibiotic antibiofilm activity against *P. aeruginosa* PAO1. Thyme EO and tobramycin were loaded on NAC to form the complex NAC(EOT + TB), covered by archeolipids extracted from *Halorubrum tebenquichense* and Tween 80: archeolipids allow for preserving NAC(EOT + TB) structural features after nebulization. NAC (EOT + TB) at noncytotoxic concentrations decreases bacterial viability and enhances the disruption of established PAO1 biofilms, especially compared with free EO and antibiotics.	Perez et al. (2022)
*M. alternifolia* (tea tree), *T. vulgaris* (thyme), *S. officinalis* (Sage), *Eucalyptus* spp.	The antibacterial effects of tea tree, thyme, sage, and eucalyptus EOs were assessed on 36 *P. aeruginosa* strains isolated from hospital infections and wastewater. The MIC and the minimum biofilm eradication concentration were used to assess the effectiveness of the EOs on both planktonic (free‐floating) and attached (biofilm) bacterial cultures. Since the highest antibacterial activity in planktonic cultures was demonstrated for tea tree (0.697%) and thyme (0.349%) EOs, these two extracts were selected to evaluate their effects on *P. aeruginosa* biofilms. The determination of the minimum biofilm inhibition concentration revealed that thyme EO, being able to inhibit biofilm formation at lower concentrations, was more effective than tea tree oil EO.	Van et al. (2022)
*C. zeylanicum* (cinnamon)	The cinnamon EO, mainly containing *trans*‐cinnamaldehyde (55.28%), *trans*‐cinnamyl acetate (12.55%), and β‐phellandrene, showed strong antibacterial activity against multidrug‐resistant *P. aeruginosa*. The EO inhibited 100% of clinical isolates of *P. aeruginosa* strains, producing a wide inhibition halo (24.72 mm in diameter). Moreover, EO showed a low MIC (0.05% v/v), outperforming the efficacy of meropenem. Cinnamon EO significantly reduced bacterial RNA content and downregulated mexA and mexB, two key efflux pump genes involved in antibiotic resistance. These effects indicate that cinnamon EO exerts both direct bactericidal action and resistance‐modulating effects, suggesting potential synergistic interactions with conventional antibiotics.	Coșeriu, Vintilă, et al. (2023)
*Clinopodium nepeta* (lesser calamint), *O. vulgare* subsp. *viridulo* (green oregano), *S. officinalis* (sage), *Salvia rosmarinus* (rosemary), *Citrus bergamia* (bergamot orange), *C. limon* (lemon), *C. reticulata* (mandarin), *F. vulgare* subsp. *piperitum* (bitter fennel), *Laurus nobilis* (bay laurel), *Myrtus communis* (myrtle)	*P. aeruginosa* showed no variation in cell growth and biofilm structure at the concentrations of the 10 EOs used in the study, from 0.1 to 10 µL/mL. Concentrations higher than 10 µL/mL were not considered because, to allow dispersion in the aqueous medium, the EOs were pre‐absorbed onto inulin; at higher concentrations, they do not dissolve completely.	D'Aquila et al. (2023)
*Zingiber officinale* (ginger)	Ginger EO showed an antimicrobial and antibiofilm effect on a multispecies biofilm composed of *Listeria monocytogenes, Salmonella typhimurium*, and *P. aeruginosa*. Once applied at a concentration of 50–100 mg/mL, it significantly reduced sessile cell counts and inhibited biofilm formation for up to 96 h. The EO disrupts the cell membrane and alters extracellular polymeric substances, thus impairing bacterial adhesion and biofilm structure.	Dos Santos et al. (2023)
*Tagetes minuta* (wild marigold)	The effect of the EO extracted from *T. minuta*, rich in *cis*‐β‐ocimene, *trans*‐tagetenone, and *cis*‐tagetenone, was evaluated against *P. aeruginosa* PAO1. The EO significantly inhibited the expression of key QS–regulated virulence factors, including pyocyanin production, protease activity, and swarming motility. Moreover, at low concentrations (20 µg/mL), the EO effectively reduced biofilm formation, suggesting that its antibiofilm activity primarily results from the disruption of QS communication pathways rather than from direct bactericidal effects.	P. Khan, Waheed, et al. (2023)
*Syzygium aromaticum* (clove)	This work describes the development of a nanoscale emulsion, using pseudo‐ternary phase diagrams, of clove EO, reported for its antibiofilm activity, loaded with levofloxacin, to enhance the antibacterial and antibiofilm activity against *P. aeruginosa* PAO1. Antibacterial activity was demonstrated, with a 16‐fold reduction in the MIC of the antibiotic against *P. aeruginosa* and an eightfold reduction against *Escherichia coli* and *Klebsiella pneumoniae* after the combined treatment. The nanoemulsion containing the antibiotic and the EO eradicated preformed *P. aeruginosa* biofilm, disrupting the biofilm matrix, causing marked cell death, and inhibiting QS activity.	Razdan et al. (2023)
*M. alternifolia* (tea tree), *Salvia sclarea* (clary sage)	*M. alternifolia* and *S. sclarea* showed a MIC of 1.25 and 5 µL/mL, respectively, against *P. aeruginosa* strains, causing a significant reduction in swarming and swimming motility at sublethal concentrations. At the same time, the two EOs reduced biofilm formation and pyocyanin production. The EOs reduced biofilm by up to 60% and inhibited protease activity by 50%. Gene expression analyses (RT‐qPCR) revealed a 50%–70% downregulation of the QS–regulated genes *lasI*, *rhlI*, and *lasR*.	Srivastava et al. (2023)
*S. aromaticum* (clove)	Clove EO, composed predominantly of carvacrol (93%) and, to a lesser extent, eugenol, *p*‑cymene, and thymol (1%, 0.8%, and 0.6%, respectively), was tested on multidrug‑resistant *P. aeruginosa* strains isolated from wound‑infected patients at Ramadi Hospital, Iraq. At concentrations of 0.00102 and 0.0005 mg/mL, a reduction in the expression of pelF, involved in bacterial biofilm formation, and aprA, encoding proteases, genes was observed. Since both genes are key virulence determinants, these results indicate that clove EO inhibits fundamental virulence mechanisms.	Awad et al. (2024)
*Cinnamomum cassia* (Chinese cinnamon), *S. aromaticum* (clove)	In this study, the EOs of *C. cassia* (CCEO) and *S. aromaticum* (CEO), known for their natural antimicrobial properties, were analyzed. GC–MS analysis revealed that the main component of CEO was eugenol (82.31%), while cinnamaldehyde (88.18%) predominates in CCEO. Both EOs and their major constituents showed good antimicrobial activity against clinical strains of *P. aeruginosa*.	Benaissa et al. (2024)
	In particular, cinnamaldehyde was the most effective, with a MIC as low as 0.031% ± 0.07% (v/v) and inhibition zones up to 30 ± 0.5 mm. Antibiofilm tests confirmed that CCEO and cinnamaldehyde were more active than CEO and eugenol, significantly reducing biofilm formation in several clinical strains. Scanning electron microscopy revealed a marked decrease in biofilm structures and deformed or shrunken bacterial cells following EOs treatment.	
*R. officinalis* (rosemary), *L. angustifolia* (lavender), *S. officinalis* (sage)	The efficacy of rosemary, lavender, and sage EOs was evaluated against both ATCC and clinical strains of *P. aeruginosa* and *K. pneumoniae*. GC–MS analysis revealed 1,8‐cineole (47.6%) as the main compound in rosemary EO, 1‐dodecene (32.65%) and linalool (29.51%) in lavender EO, and *cis*‐thujone (24.98%) and camphor (22.28%) in sage EO. All EOs exhibited antibacterial activity, with rosemary showing the strongest effect, particularly against *K. pneumoniae* ATCC. *P. aeruginosa* was resistant to lavender and sage. MIC and MBC assays confirmed the highest potency for rosemary EO (MIC 32.25–64.5 µL/mL; MBC 125 µL/mL), followed by lavender and sage. The combination of EOs with ineffective antibiotics (ceftriaxone and aztreonam) enhanced the inhibition zones against resistant *K. pneumoniae*, indicating a synergistic effect. Molecular docking analysis showed that *cis*‐thujone displayed the strongest binding affinity to bacterial target proteins, suggesting a possible mechanism for the observed antibacterial action.	Mourabiti et al. (2024)
*F. vulgare* (fennel), *C. nepeta* (nepitella), *O. vulgare* (oreganum), *S. officinalis* (sage), *S. rosmarinus* (rosemary), *L. nobilis* (bay laurel), *M. communis* (myrtle), *C. bergamia* (bergamot orange), *C. limon* (lemon), *C. reticulata* (mandarin)	Ten EOs extracted from Calabrian aromatic plants were tested alone and in combinations with antibiotics against *P. aeruginosa*. Only EO extracted from *O. vulgare* showed MIC = 5 mg/mL, while others exceeded the MIC level of 5 mg/mL, indicating limited standalone efficacy. However, several EO‐antibiotic combinations significantly enhanced antibiotic activity. For example, *C. bergamia* in combination with tetracycline produced a fourfold reduction in MIC, and all EOs combined with this antibiotic yielded 65%–75% biofilm reduction in *P. aeruginosa*. Combinations also induced distinct patterns of DNA methylation, suggesting modulation of bacterial epigenetic responses involved in resistance.	Sena et al. (2024)
*T. vulgaris* (thyme)	The EO extracted from *T. vulgaris*, characterized by high levels of thymol, *p*‐cymene, and γ‐terpinene, was evaluated across diverse genetic strains of *P. aeruginosa*. The results evidenced a striking strain‐specific variability with MIC showing differences up to 1000‐fold. Biofilm susceptibility ranged from full tolerance to near complete eradication, highlighting the heterogeneity of the response of *P. aeruginosa*. Overall, TEO demonstrated superior antipseudomonal activity compared with polyhexanide in 90% of strains. Low‐metabolism, matrix‐rich biofilms showed reduced responsiveness, suggesting that EO efficiency is influenced by both metabolic and structural factors.	Brożyna et al. (2025)
*Daucus nebrodensis* (Sicilian Wild Carrot)	The EO of *D. nebrodensis* Strobl. was subjected to chemical and biological investigations. The EO was analyzed by GC–MS and was found to be rich in monoterpene hydrocarbons, with sabinene (33.6%), α‐pinene (17.2%), γ‐terpinene (9.8%), and α‐terpinene (7.6%). The efficacy of the EO was assessed against both Gram‐negative (*E. coli* DH5α and *P. aeruginosa* PAO1), and Gram‐positive (*S. aureus* ATCC6538P, *B. subtilis* AZ54, and *M. smegmatis* MC2155) species showing significant antibacterial activity. The MIC for *B. subtilis*, the most sensitive strain, was 18 mg/mL, while for *P. aeruginosa*, the least sensitive strain, it was 30 mg/mL. Additionally, 55% inhibition of biofilm formation was observed against *M. smegmatis*.	Castagliuolo et al. (2025)
*Allium caepa* (garlic), *F. vulgare* (Fennel), *C. zeylanicum* (cinnamon), *S. aromaticum* (clove), *Artemisia pallens* (davana), *Cymbopogon martinii* (palmarosa), *T. vulgaris* (thyme)	Garlic, thyme and cinnamon EOs attenuated virulence determinants in the multidrug‐resistant pyomelanogenic *P. aeruginosa* U804 isolated from a urine sample. Pyomelanin production was reduced by treatment with garlic, thyme, and cinnamon EOs (at 0.5%) by 78.7%, 79%, and 75.7%, respectively, while biofilm formation decreased by 54%, 79%, and 59% due to the decrease of QS gene expression and in the autoinducer levels. Exposure to sub‐MIC levels of the two EOs increased outer membrane permeability, coupled with inhibition of efflux pump activity and increased intracellular retention of toxic compounds. The EO also downregulated QS–related genes (rhlI, pqsA, gacA, vfr) and reduced the amount of autoinducer. Clove EO showed medium inhibition of pyomelanin production and biofilm formation at concentrations ranging from 0.01% to 0.5% in *P. aeruginosa* strain U804s. On the contrary, Fennel, davana, and palmarosa EOs exhibited weak activity against pyomelanin synthesis on *P. aeruginosa* U804 isolated from urine samples at all concentrations ranging from 0.01% to 0.5%. Exposure of *P. aeruginosa* with 0.25% thyme, 0.06% cinnamon, and 0.125% garlic EOs before infecting *Caenorhabditis elegans* increases the nematode's survival rate.	Haripriyan et al. (2025)
*Elettaria cardamomum* var. *minuscula* (green cardamom)	The EO of *E. cardamomum* var. *minuscula*, whose chemical composition is dominated by α‐terpinyl acetate (approximately 40.9%) and 1,8‐cineole (36.2%), led to a significant reduction in the growth of *P. aeruginosa*, with biofilm inhibition reaching 97.33%. Moreover, the EO exhibited strong antimicrobial activity against Gram‐positive bacteria and yeasts. Its efficacy was confirmed both in vitro, through agar diffusion tests, and on fruits and vegetables via oil vapors. In particular, the EO in vapor form proved effective in reducing microbial loads in minimally processed food products, suggesting a promising role as a noncontact preservation method, ideal for minimally treated and vacuum‐packed foods.	Kačániová et al. (2025)
*O. vulgare* (oregano)	Zein‐tartaric acid hydrogel encapsulating oregano EO was tested for antibacterial and antibiofilm activity against *P. aeruginosa* PAO1. The formulation composed by 0.75% w/v zein + 0.75% w/v tartaric acid showed a loading efficiency of 47% for the EO. The sustained release of EO from the hydrogel reached approximately 80% within the first 30 h. At sublethal concentrations, the hydrogel reduced biofilm formation and QS activity, as evidenced by the downregulation of the lasI gene. The formulation zein‐tartaric acid hydrogel displayed higher antibiofilm activity compared with other hydrogel variants, although slightly lower than free OEO. The hydrogel retained 67% antioxidant capacity, compared with 77% for free OEO, thereby maintaining bioactivity. Cytotoxicity tests indicated low toxicity, suggesting good biocompatibility for potential biomedical applications. The encapsulated EO inhibited bacterial virulence factors acting on QS pathways.	Marzban et al. (2025)
*Lavandula spica* (spike lavender)	Spike lavender EO was extracted and characterized for its composition, revealing the presence of 28 components and the abundance of monoterpenes. The effect of EO on 56 strains of *P. aeruginosa* and on 67 of *S. aureus* (among which 30 and 38, respectively, were multidrug resistant) isolated from burn wound infections was assessed. The lavender EO showed higher growth inhibition against *S. aureus* (MIC = 0.894) than against *P. aeruginosa* (MIC = 0.818).	Siddique et al. (2025)

Abbreviations: ATCC, American Type Culture Collection; CCEO, cinnamon cassia essential oil; CEO, clove essential oil; EOT, vulgaris essential oil; FGF‐2, fibroblast growth factor‐2; GC‐MS, gas chromatography‐mass spectrometry; IL, interleukin; MIC, minimum inhibitory concentration; TB, tobramycin; THP‐1, Tohoku Hospital Pediatrics‐1; TNF, tumor necrosis factor; VEGF, vascular endothelial growth factor.

Due to their antimicrobial activity and their chemical composition rich in monoterpenes, phenolic compounds and aldehydes, known to affect membrane integrity and bacterial communication systems, the most frequently investigated EOs include *Thymus vulgaris* (Haripriyan et al. 2025; Perez et al. 2022; Brożyna et al. 2022; Van et al. 2022), *Melaleuca alternifolia* (Noumi et al. 2018; Brożyna et al. 2022; Van et al. 2022; Srivastava et al. 2023), *R. officinalis* (Ghaderi et al. 2020; Ben‐Khalifa et al. 2021; Brożyna et al. 2022; Mourabiti et al. 2024), *Syzygium aromaticum* (Haripriyan et al. 2025; Razdan et al. 2023), *Cinnamomum* spp. (Haripriyan et al. 2025; Benaissa et al. 2024; Antunes et al. 2021), *Lavandula* spp. (Donadu et al. 2018; Ghaderi et al. 2020; Mourabiti et al. 2024; Siddique et al. 2025), *Ocimum basilicum* (Pejčić et al. 2020; Brożyna et al. 2022), and the *Citrus* group (Luciardi et al. 2016; Artini et al. 2018; Luciardi et al. 2021; D'Almeida et al. 2022; D'Aquila et al. 2023).

*Cinnamomum* spp. exhibited the strongest antibacterial and antivirulence effects with MICs as 0.031% (v/v) and marked antibiofilm activity (Benaissa et al. 2024). Cinnamaldehyde, its principal component, suppressed QS–regulated genes (lasI, rhlI, pqsA, gacA, and vfr) and reduced pyocyanin and biofilm formation (Haripriyan et al. 2025).

*S. aromaticum* also demonstrated antivirulence effects on *P. aeruginosa* by downregulating pelF and aprA, which are essential genes for biofilm production and protease activity (Awad et al. 2024). Furthermore, in nanoemulsion form loaded also with levofloxacin, clove oil decreased the MIC of the antibiotic and disrupted established biofilms (Razdan et al. 2023).

*T. vulgaris* and *Coridothymus capitatus* showed consistent antibiofilm and anti‐QS activity. Thyme EO reduced biofilm formation by up to 79% and pyomelanin production by 78% (Haripriyan et al. 2025), downregulated the expression of lasI, rhlI, and vfr (Vrenna et al. 2021), and displayed antioxidant properties (Pandur et al. 2022). *C. capitatus* inhibited pyocyanin synthesis by 84%–100% and impaired bacterial motility (Vrenna et al. 2021), confirming its strong antivirulence profile.

The EO of *R. officinalis* exhibits marked antibacterial activity both alone and in combination with antibiotics. Its nanoemulsion formulation enhanced efficacy by approximately four‐ to eightfold compared with the bulk form (Ghaderi et al. 2020), and it showed a strong ability to inhibit *P. aeruginosa* biofilm formation (Ghaderi et al. 2020; Brożyna et al. 2022). Co‐encapsulation with cefepime in lipid nanoparticles further increased antibacterial activity against cefepime‐resistant strains (Ben‐Khalifa et al. 2021). Moreover, when its main component, 1,8‐cineole, was compared with the EOs of *Lavandula angustifolia* and *S. officinalis*, it yielded the lowest MIC values (32.25–64.5 µL/mL) and MBC values (125 µL/mL) against both ATCC and clinical isolates of *P. aeruginosa* (Mourabiti et al. 2024).

Similarly, *O. basilicum* and *S. officinalis* proved in vitro biofilm inhibition rates of up to 99% and suppression of lasI and rhlI QS systems. In addition, both EOs reduced twitching, swimming, and swarming motility (Pejčić et al. 2020).

Other promising EOs include *Satureja khuzistanica*, which acts by reducing the expression of efflux pump genes MexE and MexY, thereby increasing the effectiveness of gentamicin and norfloxacin (Iman Islamieh et al. 2020). *Elettaria cardamomum* var. *minuscula* achieved remarkable antibiofilm inhibition of 97.33% and exhibited antimicrobial activity against Gram‐positive bacteria and yeast (Kačániová et al. 2025). *M. piperita* also appears to play a role against *P. aeruginosa*, as its EO at sub‐MIC levels reduced the enzymatic activities of elastase, protease, and chitinase, decreased pyocyanin and exopolysaccharide production, and markedly inhibited cell–cell communication through QS. Its activity seems to be mainly related to its ability to bind the LasR receptor and compete with the AHL signal, which is essential for the QS system (Husain et al. 2015).

Considering the *Citrus* genus, it is possible to group together a series of studies forming a “*Citrus* group,” which includes *Citrus limon* (Artini et al. 2018; Luciardi et al. 2021), *C. bergamia* (D'Aquila et al. 2023), *Citrus reticulata* (Luciardi et al. 2016; D'Almeida et al. 2022), and *Citrus paradisi* (D'Almeida et al. 2022). Studies have shown contrasting results. *Citrus* EOs, and in particular lemon EO, lemon terpenes, and lemon essence, contain high amounts of monoterpene hydrocarbons, with limonene as the main component. These EOs induced inhibition of biofilm formation, reduction of metabolic activity, reduction of elastase activity, and reduction of swarming motility in *P. aeruginosa* (Luciardi et al. 2021; D'Almeida et al. 2022). Several studies report the reduction of NAHL signal molecule production as a factor associated with the reduction of cellular functions of *P. aeruginosa* (Luciardi et al. 2016, 2021). The use of limonene alone, however, does not seem to have much effect (Luciardi et al. 2021; D'Almeida et al. 2022), and in some cases it even seems to favor biofilm formation (Artini et al. 2018). Other studies have not produced significant results, such as that of D'Aquila et al. (2023).

The EO of *M. alternifolia* leads to a reduction in swarming and swimming motility in *P. aeruginosa*, as well as a decrease in biofilm formation and pyocyanin production, which is crucial for QS (Noumi et al. 2018; Srivastava et al. 2023).

Altogether, these findings highlight the promising potential use of EOs alone or in combination with antibiotics, particularly against *P. aeruginosa* antibiotic‐resistant strains. However, it should be noted that most available studies have been conducted exclusively in vitro, and their therapeutic potential remains to be evaluated in vivo. Moreover, the variable chemical composition of EOs, caused by plant origin, harvesting season, extraction methods, and storage conditions, may complicate the standardization of their effects. Finally, besides these EOs, which are the most frequently cited, studied, and used, there are many others, less investigated, that are potentially effective against P. aeruginosa.

#### 5.2.1 Quality and Limitations of Current Evidence

Despite the large number of studies reporting antimicrobial, antibiofilm, and anti‐virulence activities of EOs against *P. aeruginosa*, the overall quality and comparability of the available evidence remain limited.

One of the major sources of heterogeneity is the lack of methodological standardization. Studies employ different bacterial strains, including reference strains (e.g., PAO1 and ATCC 27853) and clinical multidrug‐resistant isolates, which may differ substantially in susceptibility profiles and virulence traits. In addition, antimicrobial activity is assessed using a variety of experimental approaches, including broth microdilution, agar diffusion assays, biofilm inhibition assays, and QS inhibition tests, often under different culture conditions and using different growth media, solvents, and incubation parameters.

Antimicrobial efficacy is also highly variable. MICs are reported in different units (e.g., % v/v, μg/mL, mg/mL, and μL/mL), making direct quantitative comparisons difficult. Furthermore, many studies evaluate whole EOs, whereas others focus on purified compounds, such as cinnamaldehyde, thymol, carvacrol, eugenol, or limonene. Because EO composition is strongly influenced by plant genotype, geographical origin, harvesting period, extraction method, and storage conditions, studies investigating oils from the same plant species may report markedly different chemical profiles and biological activities.

Another important limitation is that most studies rely exclusively on in vitro models. Although inhibition of bacterial growth, QS, virulence‐factor production, and biofilm formation is frequently reported, experimental conditions often do not reproduce the complexity of clinical infections. Mature biofilms, host immune responses, pharmacokinetic constraints, and tissue penetration are rarely considered. In addition, only a minority of studies include cytotoxicity assessments or evaluate the therapeutic window between antimicrobial efficacy and host–cell toxicity.

Contradictory findings are also observed for some EOs. *Citrus*‐derived oils provide a representative example: while several studies reported significant reductions in biofilm formation, QS activity, and virulence‐factor production, others observed limited or no significant antimicrobial effects. Similarly, limonene, often identified as the major active constituent of *Citrus* oils, showed weaker activity than the complete EO and, in some cases, even promoted biofilm formation. These discrepancies suggest that synergistic interactions among EO constituents may be more important than the activity of individual compounds and highlight the need for standardized chemical characterization.

Overall, the current body of evidence supports the potential of EOs as antimicrobial and anti‐virulence agents against *P. aeruginosa*. However, differences in experimental design, EO composition, bacterial strains, outcome measurements, and reporting criteria limit cross‐study comparisons and prevent robust conclusions regarding relative efficacy. Future studies should adopt harmonized susceptibility‐testing protocols, standardized chemical characterization, clinically relevant infection models, and transparent reporting practices to improve reproducibility and facilitate translation into therapeutic applications.

## 6 In Vivo Studies on Animal Models

Although several studies have demonstrated the antimicrobial efficacy of EOs against human clinical isolates of *P. aeruginosa*, robust clinical evidence in humans remains very limited. To date, no well‐designed clinical trials specifically evaluating EOs as therapeutic agents against *P. aeruginosa* infections are available. Most translational evidence derives from topical applications, wound‐healing models, or studies targeting mixed microbial infections rather than *P. aeruginosa* infections specifically. Only a limited number of mammalian studies, mainly in rodent models, have been conducted, and these generally report local effects, relying on topical administration, rather than complete eradication of infection. These investigations typically focus on topical applications and assess both wound‐healing parameters—such as bacterial clearance, inflammation, collagen synthesis, and re‐epithelialization—and molecular markers associated with inflammation, angiogenesis, and tissue regeneration. As an example, the efficacy of an ointment containing *Mentha piperita* EO on the healing of wounds infected by *S. aureus* and *P. aeruginosa* was assessed in infected mice. A decline of the bacterial density inside the tissue and a reduction of the edema and inflammation level, coupled with an enhancement of fibroblast migration, collagen production and re‐epithelization was observed. Moreover, an up regulation of genes encoding for cytokines, interleuchin and those involved in fibroblast activation was observed in animals treated with the EO. In parallel, a downregulation of the expression of the genes encoding TNF‐alpha, playing a major role in the first stage of the inflammatory phase, and of the vascular endothelial growth factor (VEGF) and fibroblast growth factor‐2 (FGF‐2), promoting angiogenesis during wound healing, was measured. Although these interesting results, the mechanisms underlying this double action are unclear (Modarresi et al. 2019).

An evolution of the use of the EOs oil in in vivo experiments is the encapsulation in nanocarrier systems that can protect volatile and unstable components from degradation, improving their chemical stability, allowing controlled release, and reducing cytotoxicity. In this context, Khezri et al. (2019) assessed the antibacterial effect of rosemary EO by using nanostructured lipid carriers. This research was performed in vivo, contaminating wounds in mice with ATCC standard of *S. aureus*, *Staphylococcus epidermidis*, *Streptococcus pneumoniae*, *E. coli*, *Bacillus anthracis*, *Listeria monocytogenes*, *Salmonella typhimurium*, and *P. aeruginosa*. The results showed that the encapsulation did not affect the in vitro efficiency of the EO. While the EO inhibited the growth of the considered bacterial species in vitro, the conventional antibiotics (gentamicin, penicillin, and mupirocin) showed the highest rate of inhibition. A reduction of the bacterial density and of the wound size in mice, coupled with enhanced vascularization, fibroblast infiltration, re‐epithelialization rate, collagen synthesis and the serum levels of IL‐3, IL‐10, VEGF and SDF‐1 alpha serum levels were observed and clearly indicated their potential uses of encapsulated EOs for the treatment of infected wounds.

Similar results were obtained 1 year later by Farahpour et al. (2020), who evaluated the effects of an ointment containing *Salvia officinalis* EO, whose main constituents were *cis*‐thujone, camphor, *trans*‐thujone, and 1,8‐cineole, on mice wounds infected with *P. aeruginosa* and *S. aureus*. Again, the inflammation phase was reduced, while the cellular proliferation, the collagen production, and re‐epithelialization were boosted by EO treatment. Molecular analysis allowed detection of increased mRNA levels of FGF‐2 and VEGF, and up‐regulation of cyclin‐D1 and Bcl‐2 in animals treated topically with *S. officinalis* EO.

The effect of topical administration of an ointment containing EOs extracted from *Satureja sahendica*, an Iran native plant species with anti‐inflammatory properties, was assessed in BALB/c mices. Wounds in mice were infected with *P. aeruginosa* and *S. aureus* and treated with the antibiotic mupirocin or with the ointment containing 1%, 2%, and 4% of the EO. The main results obtained in this work was a shorter period of inflammation of the infected wounds treated with EO, coupled with increased cell proliferation rate, enhanced level of the collagen deposition and faster rehepithelialization. Molecular analysis showed that the expressions of insulin‐like growth factor (IGF)‐1, FGF‐2, VEGF, transforming growth factor beta (TGF‐beta), and chemokine (CXC motif) ligand 1 (CXCL‐1) genes were upregulated in mice treated topically compared with control animals treated with soft yellow paraffin (Omarizadeh et al. 2021).

More recently, the wound‐healing properties of *Achillea millefolium* EO were assessed in rats. Excisional wounds were created on the animals, infected with *S. aureus* and *P. aeruginosa*, and treated topically with an ointment containing different concentrations of EO (1%, 2%, and 3%). Topical application not only reduced bacterial density, but also inflammation and edema, and increased collagen accumulation, further confirming the wound‐healing potential of EOs in polymicrobial infections (Ghasemi et al. 2023).

Collectively, these findings indicate that EOs—especially when formulated in nanocarrier systems—can modulate both microbial dynamics and host repair responses in *P. aeruginosa*‐associated wound infections. However, the limited number of studies, the variability in experimental design, and the exclusive focus on topical models highlight the need for more systematic and mechanistic investigations to fully elucidate their therapeutic potential.

## 7 Toxicity and Safety Considerations

### 7.1 Cytotoxicity Studies

The pronounced lipophilicity and complex chemical composition of EOs enable pleiotropic effects, allowing simultaneous interactions with multiple cellular targets. As stated before, in bacteria, EOs disrupt the cell wall and cytoplasmic membrane, causing ion leakage, collapse of membrane potential, inhibition of proton pumps, ATP depletion, and denaturation of lipids and proteins, ultimately leading to cell lysis. However, since these mechanisms are not strictly selective for prokaryotic cells, similar interactions may occur in host tissues when concentrations exceed safe thresholds, thus leading to cytotoxic effects on eukaryotic cells. In fact, their main mechanism in eukaryotic cells is to behave as pro‐oxidant agents, affecting intracellular membranes, particularly those of mitochondria. This results in mitochondrial depolarization, calcium imbalance, reduced pH gradient, and energy deficits, triggering reactive oxygen species production, cytochrome c release, and activation of apoptotic or necrotic pathways. In addition, ultrastructural analyses revealed membrane damage, cytoplasmic swelling, and nuclear alterations, while lipidomic studies indicated a decrease in unsaturated fatty acids and an increase in saturated ones, reflecting reduced membrane fluidity and structural stability (Bakkali et al. 2008).

Another mechanism by which EOs can induce cytotoxicity is the promotion of caspase‐mediated apoptosis, evidenced by phosphatidylserine externalization. Both intrinsic and extrinsic apoptotic pathways are involved, with preferential targeting of tumor or precancerous cells (Sœur et al. 2011). Overall, these findings indicate that EOs exert multitarget cytotoxic effects via pro‐oxidant mechanisms and structural and metabolic alterations, influencing apoptosis, cell‐cycle progression, and embryonic development. These data support their therapeutic potential, while highlighting the critical importance of careful dose regulation to minimize undesired toxic effects (Gautam et al. 2014).

Other cytotoxic effects arise from apoptotic processes, as demonstrated by a concentration‐dependent reduction in mitochondrial membrane potential following exposure to EOs. At the molecular level, increased expression of cytochrome c, Bax, caspase‐9, and caspase‐3 is observed, while Bcl‐2 is decreased, indicating activation of the mitochondrial apoptotic pathway. This effect occurs following exposure to *Chenopodium ambrosioides* EO, which also induces S‐phase cell‐cycle arrest in L02 cells, as revealed by flow cytometry analysis (Wang et al. 2021).

Experimental studies in zebrafish embryos have shown that exposure to various EOs significantly impacts embryonic development, causing delayed epiboly, morphometric and behavioral alterations, and increased mortality. LC50 values were determined to be 3.7 µg/mL for citronella oil, 14.4 µg/mL for thyme oil, and 5.3 µg/mL for oregano oil. Exposed larvae exhibited growth retardation, spinal deformities, pericardial edema, smaller eyes, and impaired swim bladder inflation, while behavioral assessments revealed altered swimming activity during light–dark cycles, suggesting potential neurotoxic effects during development (da Silva et al. 2023).

Recently, the EO of *Lippia alba*, comprising 36 constituents, with carvacrol, *p*‐cymene, γ‐terpinene, and thymol as the predominant compounds, demonstrated marked antimicrobial activity against multidrug‐resistant clinical isolates of another Gram‐negative opportunistic pathogen, *A. baumannii*, with MIC values ranging from 64 to 128 µg/mL and MBC values that were largely comparable, indicating a primarily bactericidal mode of action. A combination of the EO with imipenem and gentamicin yielded additive or synergistic interactions, suggesting a potential adjuvant role. Cytotoxicity assessment using the colorimetric methylthiazolyltetrazolium salt assay on mammalian peritoneal macrophages, revealing the cell metabolic activity, showed a half maximal cytotoxic concentration (CC_50_) exceeding 512 µg/mL, a value substantially higher compared with the antimicrobial concentrations, thus indicating low cellular toxicity coupled to minimal hemolytic activity (< 2% at inhibitory concentrations). Overall, EO extracted from *L. alba* displays a favorable risk–benefit profile, characterized by strong activity against multidrug‐resistant isolates and low cytotoxicity toward mammalian cells, supporting its potential application as a natural antimicrobial agent or as an adjuvant to conventional antibiotics (Ferreira de Santana et al. 2024).

Finally, chronic exposure to cinnamaldehyde, the main constituent of cinnamon EO, can unexpectedly shape antibiotic resistance in *P. aeruginosa*. This concept was first demonstrated by Tetard et al. (2022), who showed that repeated exposure of *P. aeruginosa* to subinhibitory concentrations of cinnamaldehyde quickly selected mutants with increased tolerance to this compound and concomitant MDR to clinically relevant antibiotics. Whole‐genome sequencing of the bacterial strains revealed recurrent mutations in nalC, a negative regulator of the MexAB–OprM efflux system, leading to its overexpression and cross‐resistance to β‐lactams and fluoroquinolones. More recently, Dubois et al. (2025) extended this work; the authors identified mutations affecting the ATP synthase complex or its regulation, resulting in altered respiratory activity and proton motive force after cinnamaldehyde treatment. This mechanism contributes to cinnamaldehyde tolerance and also induces unexpected hypersusceptibility to aminoglycosides and colistin, which may be related to enhanced antibiotic uptake. Together, these two studies reveal that adaptation to cinnamaldehyde does not merely select for stable MDR but can modulate *P. aeruginosa* physiology, leading to susceptibility profiles with potential clinical implications. The potential benefits and risks of combining EOs or their components with conventional antibiotics need careful evaluation, including the determination of their effective concentrations in vivo. In particular, assessing whether electrophilic agents like cinnamaldehyde can induce the rise of genetically unstable subpopulations in CF‐affected patients colonized by *P. aeruginosa* would carry significant clinical implications.

### 7.2 Challenges and Limitations, Potential Side Effects, and Contraindications

Despite the growing interest in EOs as natural antimicrobial and anti‐virulence agents, several critical challenges and limitations currently hinder their translation into clinical practice against human opportunistic pathogens, such as *P. aeruginosa*. To date, no clinical evidence in humans specifically supports the use of EOs for the treatment of *P. aeruginosa* infections. The available literature is largely restricted to in vitro studies or investigations conducted on clinical isolates or ATCC reference strains, which do not fully capture the complexity of host–pathogen interactions, immune responses, or the pharmacological constraints encountered in vivo. Moreover, even when promising antimicrobial, antibiofilm, or anti‐QS effects are reported, these outcomes are often achieved at concentrations that may not be clinically achievable or safe.

A major limitation in the development of EO‐based therapeutics derives from their intrinsic chemical variability. EO composition is strongly influenced by plant species, plant organ, geographical origin, harvesting season, and extraction method, resulting in marked heterogeneity across batches. This variability complicates standardization, reproducibility, and dose optimization, which are essential prerequisites for pharmaceutical development. Furthermore, most experimental studies rely on simplified models, frequently focusing on mono‐species biofilms or acute infection settings, whereas *P. aeruginosa* infections in clinical contexts are often chronic, polymicrobial, and associated with complex and highly structured biofilm architectures.

Safety concerns further limit the translational potential of EOs. Although often perceived as inherently safe due to their natural origin, many EO constituents—such as phenols, aldehydes, and certain terpenes—can exert cytotoxic, irritant, or pro‐inflammatory effects at higher doses. Data on toxicity, pharmacokinetics, metabolism, and long‐term exposure remain scarce, and potential interactions with conventional antibiotics or host detoxification pathways, particularly cytochrome P450 enzymes, are poorly characterized (Hammer et al. 2006). These gaps raise concerns regarding systemic administration and largely restrict current experimental and potential clinical applications of EOs to topical or localized use.

Contraindications should also be carefully considered. Several EOs are not recommended for vulnerable populations, including pregnant women, neonates, elderly individuals, or patients with respiratory or neurological disorders, due to reported neurotoxic, allergenic, or irritant effects. These risks substantially limit systemic applications and further emphasize the need for caution when considering clinical use.

Experimental and clinical evidence increasingly challenge the assumption that EOs are inherently safe. Toxicological studies and clinical case reports demonstrate that ingestion of certain EOs can induce severe neurological, respiratory, and multiorgan toxicity, with manifestations, including seizures, central nervous system depression, metabolic acidosis, aspiration pneumonitis, and, in extreme cases, fatal outcomes (Hammer et al. 2006; Suresh et al. 2025). These observations indicate that systemic toxicity is not merely theoretical and that toxic dose thresholds may be relatively low and highly variable among individuals.

Consistent with these findings, a retrospective analysis of EO poisoning cases reported to a national poison center over more than a decade documented a substantial incidence of clinically relevant adverse effects across different age groups, including infants, children, and adults (Belghiti et al. 2023). Reported effects ranged from mild and transient symptoms—such as nausea, headache, dizziness, fatigue, and cutaneous or labial irritation—to more severe manifestations requiring hospitalization, including convulsive seizures. Notably, the majority of cases were associated with therapeutic use rather than accidental exposure, indicating that inappropriate dosing, lack of dilution, and repeated or prolonged use represent major risk factors (Belghiti et al. 2023). Several commonly used EOs, including lavender, eucalyptus, rosemary, spearmint, and white wormwood, were implicated, with neurotoxic effects attributed to specific constituents such as thujone, cineole, pulegone, and other monoterpenic ketones known to act on the central nervous system. In pediatric cases, topical application of undiluted oils or prolonged inhalation emerged as particularly critical exposure routes, highlighting the increased vulnerability of this population (Belghiti et al. 2023).

Topical administration, often regarded as a safer route of exposure, is also associated with nonnegligible risks. Numerous EOs and their oxidized constituents have been repeatedly linked to irritant and allergic contact dermatitis, photoallergic reactions, and inflammatory skin responses (Hammer et al. 2006; Gnaneswaran et al. 2025). Lavender EO, one of the most extensively studied EOs, exemplifies this duality: while it has demonstrated antimicrobial and anti‐inflammatory activity and a potential role in wound healing, its clinical applicability is limited by well‐documented allergic contact dermatitis and photoallergic reactions, particularly associated with oxidized constituents, such as linalool and linalyl acetate (Gnaneswaran et al. 2025).

Other EOs, including frankincense and geranium oils, have shown antimicrobial, anti‐inflammatory, antioxidant, and wound‐healing properties in experimental models, with additional evidence suggesting effects on fibroblast proliferation and tissue remodeling (Gnaneswaran et al. 2025). However, clinical data remain limited, and isolated reports of tissue damage associated with inappropriate topical use highlight the risks linked to misinformation and self‐treatment. Similarly, neroli and patchouli oils further illustrate the dual nature of these compounds: while neroli oil has demonstrated antimicrobial activity against Gram‐negative bacteria, including *P. aeruginosa*, and anti‐inflammatory effects in vitro, it has been associated with contact dermatitis and photoallergic reactions, particularly in occupational settings, whereas patchouli oil has shown wound‐healing potential in animal models but lacks robust human evidence (Gnaneswaran et al. 2025).

Beyond local toxicity, increasing attention has been directed toward the potential endocrine‐disrupting properties of specific EOs. In vitro studies have demonstrated that components of lavender and tea tree oils exhibit estrogenic and anti‐androgenic activity through interactions with hormone receptor signaling pathways (Ramsey et al. 2019). Clinically, prolonged exposure to products containing these oils has been temporally associated with cases of premature thelarche and prepubertal gynecomastia, with regression observed following discontinuation (Ramsey et al. 2019; Braunstein and Braunstein 2023). Although dermal absorption appears limited and a direct causal relationship remains debated, these findings raise important concerns regarding chronic exposure, cumulative dosing, and susceptibility in vulnerable populations.

Finally, regulatory and translational barriers must also be considered. EOs are generally classified by regulatory agencies as cosmetic ingredients, food additives, or traditional remedies rather than pharmaceutical products, which substantially limits incentives for conducting well‐designed clinical trials (Guerriaud 2018). In the European Union, their use is primarily governed by cosmetic legislation rather than medicinal product regulations (Regulation (EC) No. 1223/2009), further constraining their development as therapeutic agents. Consequently, despite their promising antimicrobial and anti‐virulence properties, rigorous in vivo studies, standardized formulations, comprehensive toxicological assessments, and controlled clinical trials will be essential to clarify the true therapeutic potential of EOs and to define appropriate indications and contraindications.

Overall, these considerations underscore that EOs cannot be regarded as inherently safe therapeutic agents solely on the basis of their natural origin or traditional use. Comprehensive toxicological characterization, standardized formulations, and robust evidence‐based risk–benefit assessments are indispensable prerequisites before EOs can be responsibly considered for clinical application, particularly in contexts involving chronic exposure or systemic administration.

## 8 Conclusion: Potential Applications and Future Perspectives

The growing global burden of *P. aeruginosa* infections, particularly those caused by multidrug‐resistant strains, highlights the urgent need for innovative therapeutic strategies. Due to their broad‐spectrum antimicrobial activity and ability to target multiple bacterial processes simultaneously, EOs have emerged as promising candidates able to provide a multifaceted approach to suppress growth and attenuate pathogenicity in *P. aeruginosa*.

Although experimental evidence supports the direct application of EOs as alternatives or adjuvants to conventional antibiotics, translation in a clinical context remains limited due to their chemical instability, volatility, cytotoxicity, and inconsistent delivery. In vivo studies are scarce and largely confined to topical infection models, while controlled clinical trials are completely lacking. Additional challenges include chemical variability, lack of standardization, and insufficient pharmacokinetic and toxicological information. These limitations walk together with the misconception that natural products are inherently safe.

Therefore, it is clear that the development of standardized, terpene‐enriched EO formulations is crucial. By minimizing inactive or potentially harmful compounds, such formulations could achieve higher bioactivity at lower doses, thereby reducing adverse effects, improving patient safety and tolerance, and decreasing the risk of antibiotic resistance by limiting selective pressure. Nanotechnology‐based delivery systems, including liposomes, solid lipid nanoparticles, and polymeric nanocarriers, offer further advantages by protecting volatile compounds from degradation, enabling controlled and stimuli‐responsive release, enhancing solubility, and reducing cytotoxicity, while preserving antimicrobial and anti‐virulence activity. These systems also allow site‐specific delivery, maximizing therapeutic efficacy and minimizing systemic exposure.

Beyond direct antimicrobial effects, EO formulations can act as anti‐virulence agents, targeting QS, biofilm formation, and toxin production. The combined use of EOs and conventional antibiotics, potentiate the activity of the single treatment, reducing effective doses and limiting resistance evolution. Synergistic interactions between EO components—such as carvacrol, thymol, and eugenol—and antibiotics like tetracyclines or aminoglycosides further support the potential of combination therapies.

In our opinion, future research should focus on (i) developing standardized, terpene‐enriched formulations with improved safety profiles; (ii) advancing delivery strategies, including nanocarriers and stimuli‐responsive materials; and (iii) exploring EO‐based medical devices that prevent biofilm formation, modulate virulence, and promote tissue healing with minimal systemic toxicity. While EOs are not yet ready to serve as standalone therapies against *P. aeruginosa*, they are promising components of complementary strategies.

## Author Contributions

**Giorgia Novello:** writing – original draft, conceptualization, supervision. **Elisa Bona:** writing – original draft, writing – review and editing, conceptualization, supervision. **Manuel Petroselli:** writing – original draft, writing – review and editing, supervision. **Chiara Bazzano:** writing – original draft, writing – review and editing. **Stefano Chiesa:** resources, writing – original draft. **Tabata Gaggero:** resources, writing – original draft. **Giorgio Gaglio:** resources, writing – original draft. **Gabriele Magnani:** resources, writing – original draft. **Alfonso Pampaloni Pasetti:** resources, writing – original draft. **Simone Repetto:** resources, writing – original draft. **Erica Rotella:** resources, writing – original draft. **Alice Topino:** resources, writing – original draft. **Emanuela Serra:** resources, writing – original draft. **Manuel Vidali:** resources, writing – original draft. **Silvia Zonca:** resources, writing – original draft. **Elisa Gamalero:** conceptualization, writing – original draft, writing – review and editing, supervision.

## Funding

The authors have nothing to report.

## Ethics Statement

The authors have nothing to report.

## Conflicts of Interest

None declared.

## Data Availability

The authors have nothing to report.

## References

[mbo370339-bib-0001] Abbad, I. , B. Soulaimani , M. Iriti , and M. Barakate . 2025. “Chemical Composition and Synergistic Antimicrobial Effects of Essential Oils From Four Commonly Used *Satureja* Species in Combination With Two Conventional Antibiotics.” Chemistry & Biodiversity 22, no. 7: e202402093. 10.1002/cbdv.202402093. 40014760 PMC12270359

[mbo370339-bib-0002] Abo‐Zaid, G. A. , A. S. Abdullah , N. A.‐M. Soliman , et al. 2023. “Evaluation of Bio‐Friendly Formulations From Siderophore‐Producing Fluorescent *Pseudomonas* as Biocontrol Agents for the Management of Soil‐Borne Fungi, *Fusarium oxysporum* and *Rhizoctonia solani* .” Agriculture (London) 13: 1418. 10.3390/agriculture13071418.

[mbo370339-bib-0003] Al‐Helo, F. K. , N. El‐Banna , H. Qaralleh , M. O. Al‐Limoun , and K. Khleifat . 2025. “Quorum Sensing Inhibition and Virulence Factor Attenuation in *Pseudomonas aeruginosa* by Camphor.” Journal of Pharmacopuncture 8, no. 3: 229–239. 10.3831/KPI.2025.28.3.229. PMC12464080 41018868

[mbo370339-bib-0004] Alibi, S. , W. Ben Selma , J. Ramos‐Vivas , et al. 2020. “Anti‐Oxidant, Antibacterial, Anti‐Biofilm, and Anti‐Quorum Sensing Activities of Four Essential Oils Against Multidrug‐Resistant Bacterial Clinical Isolates.” Current Research in Translational Medicine 68: 59–66. 10.1016/j.retram.2020.01.001. 32192922

[mbo370339-bib-0005] Almanaa, T. N. , N. S. Alharbi , G. Ramachandran , et al. 2021. “Anti‐Biofilm Effect of *Nerium oleander* Essential Oils Against Biofilm Forming *Pseudomonas aeruginosa* on Urinary Tract Infections.” Journal of King Saud University—Science 33: 101340. 10.1016/j.jksus.2021.101340.

[mbo370339-bib-0006] Antunes, J. C. , T. D. Tavares , M. A. Teixeira , et al. 2021. “Eugenol‐Containing Essential Oils Loaded Onto Chitosan/Polyvinylalcohol Blended Films and Their Ability to Eradicate *Staphylococcus aureus* or *Pseudomonas aeruginosa* From Infected Microenvironments.” Pharmaceutics 13: 195. 10.3390/pharmaceutics13020195. 33540524 PMC7912801

[mbo370339-bib-0007] Artini, M. , A. Patsilinakos , R. Papa , et al. 2018. “Antimicrobial and Antibiofilm Activity and Machine Learning Classification Analysis of Essential Oils From Different Mediterranean Plants Against *Pseudomonas aeruginosa* .” Molecules 23: 482. 10.3390/molecules23020482. 29473844 PMC6017904

[mbo370339-bib-0008] Awad, F. , M. Mohaisen , and K. Ahmed Al‐Taee . 2024. “The Effect of Sub‐Inhibitory Concentration of Clove Essential Oil on the Expression of *Pseudomonas aeruginosa* Virulence Genes.” Tropical Journal of Natural Product Research (TJNPR) 8, no. 3: 6498–6502. 10.26538/tjnpr/v8i3.4.

[mbo370339-bib-0009] Bakkali, F. , S. Averbeck , D. Averbeck , and M. Idaomar . 2008. “Biological Effects of Essential Oils—A Review.” Food and Chemical Toxicology 46, no. 2: 446–475. 10.1016/j.fct.2007.09.106. 17996351

[mbo370339-bib-0010] Bekka‐Hadji, F. , I. Bombarda , F. Djoudi , S. Bakour , and A. Touati . 2022. “Chemical Composition and Synergistic Potential of *Mentha pulegium* L. and *Artemisia herba alba* Asso. Essential Oils and Antibiotic Against Multi‐Drug Resistant Bacteria.” Molecules 27 3: 1095. 10.3390/molecules27031095. 35164360 PMC8839733

[mbo370339-bib-0011] Belghiti, A. A. , M. Yafout , A. M. Alaoui , R. Soulaymani , A. Chebat , and A. A. H. Said . 2023. “Retrospective Study of Cases of Poisoning by Essential Oils in Morocco.” Toxicologie Analytique et Clinique 35: 133–137. 10.1016/j.toxac.2022.11.001.

[mbo370339-bib-0012] Benaissa, A. , A. Khadir , A. N. Tamfu , et al. 2024. “Biofilm Disruption and Virulence Attenuation Effects of Essential Oil From Endemic Algerian *Cistus munbyi* (Cistaceae) Against Clinical Strains of *Pseudomonas aeruginosa* .” Natural Product Communications 19, no. 4: 20. 10.1177/1934578X241245234.

[mbo370339-bib-0013] Ben‐Khalifa, R. , F. B. Gaspar , C. Pereira , L. Chekir‐Ghedira , and S. Rodríguez‐Rojo . 2021. “Essential Oil and Hydrophilic Antibiotic Co‐Encapsulation in Multiple Lipid Nanoparticles: Proof of Concept and *In Vitro* Activity Against *Pseudomonas aeruginosa* .” Antibiotics (USSR) 10: 1300. 10.3390/antibiotics10111300. PMC8614727 34827238

[mbo370339-bib-0014] Borisova, D. , T. Paunova‐Krasteva , T. Strateva , and S. Stoitsova . 2025. “Biofilm Formation of *Pseudomonas aeruginosa* in Cystic Fibrosis: Mechanisms of Persistence, Adaptation, and Pathogenesis.” Microorganisms 13: 1527. 10.3390/microorganisms13071527. 40732035 PMC12299273

[mbo370339-bib-0015] Braunstein, E. W. , and G. D. Braunstein . 2023. “Are Prepubertal Gynaecomastia and Premature Thelarche Linked to Topical Lavender and Tea Tree Oil Use?” touchREVIEWS in Endocrinology 19: 60–68. 10.17925/EE.2023.19.2.9. PMC10769481 38187077

[mbo370339-bib-0016] Brint, J. M. , and D. E. Ohman . 1995. “Synthesis of Multiple Exoproducts in *Pseudomonas aeruginosa* Is Under the Control of RhlR–RhlI, Another Set of Regulators in Strain PAO1 With Homology to the Autoinducer‐Responsive LuxR–LuxI Family.” Journal of Bacteriology 177: 7155–7163. 10.1128/jb.177.24.7155-7163.1995. 8522523 PMC177595

[mbo370339-bib-0017] Brożyna, M. , J. Paleczny , W. Kozłowska , et al. 2022. “Chemical Composition and Antibacterial Activity of Liquid and Volatile Phase of Essential Oils Against Planktonic and Biofilm‐Forming Cells of *Pseudomonas aeruginosa* .” Molecules 27: 4096. 10.3390/molecules27134096. 35807343 PMC9268626

[mbo370339-bib-0018] Brożyna, M. , Z. Stępnicka , N. Tymińska , et al. 2025. “Toward Essential Oil Stewardship: Strain‐Resolved Evaluation of Thyme Oil Activity Against *Pseudomonas aeruginosa* .” Frontiers in Pharmacology 16: 1659096. 10.3389/fphar.2025.1659096. 41079731 PMC12510957

[mbo370339-bib-0019] Castagliuolo, G. , A. Porrello , M. Cerasola , et al. 2025. “Antimicrobial Properties of *Daucus nebrodensis* Strobl.: A Multifunctional Essential Oil Against Bacterial Pathogens.” Plants 14: 2227. 10.3390/plants14142227. 40733464 PMC12298526

[mbo370339-bib-0020] Chemat, F. , M. A. Vian , and G. Cravotto . 2012. “Green Extraction of Natural Products: Concept and Principles.” International Journal of Molecular Sciences 13: 8615–8627. 10.3390/ijms13078615. 22942724 PMC3430255

[mbo370339-bib-0021] Cigana, C. , J. Castandet , N. Sprynski , et al. 2021. “ *Pseudomonas aeruginosa* Elastase Contributes to the Establishment of Chronic Lung Colonization and Modulates the Immune Response in a Murine Model.” Frontiers in Microbiology 12: 620819. 10.3389/fmicb.2020.620819. PMC7836092 33510733

[mbo370339-bib-0022] Coșeriu, R. L. , A. D. Mare , F. Toma , et al. 2023. “Uncovering the Resistance Mechanisms in Extended‐Drug‐Resistant *Pseudomonas aeruginosa* Clinical Isolates: Insights From Gene Expression and Phenotypic Tests.” Microorganisms 11: 2211. 10.3390/microorganisms11092211. 37764055 PMC10535578

[mbo370339-bib-0023] Coșeriu, R. L. , C. Vintilă , M. Pribac , et al. 2023. “Antibacterial Effect of 16 Essential Oils and Modulation of Mex Efflux Pumps Gene Expression on Multidrug‐Resistant *Pseudomonas aeruginosa* Clinical Isolates: Is Cinnamon a Good Fighter?” Antibiotics (USSR) 12: 163. 10.3390/antibiotics12010163. PMC9854426 36671364

[mbo370339-bib-0024] Crone, S. , M. Vives‐Flórez , L. Kvich , et al. 2020. “The Environmental Occurrence of *Pseudomonas aeruginosa* .” APMIS 128: 220–231. 10.1111/apm.13010. 31709616

[mbo370339-bib-0025] D'Almeida, R. E. , N. Sued , and M. E. Arena . 2022. “ *Citrus paradisi* and *Citrus reticulata* Essential Oils Interfere With *Pseudomonas aeruginosa* Quorum Sensing *In Vivo* on *Caenorhabditis elegans* .” Phytomedicine Plus 2: 100160. 10.1016/j.phyplu.2021.100160.

[mbo370339-bib-0026] D'Aquila, P. , G. Sena , M. Crudo , G. Passarino , and D. Bellizzi . 2023. “Effect of Essential Oils of Apiaceae, Lamiaceae, Lauraceae, Myrtaceae, and Rutaceae Family Plants on Growth, Biofilm Formation, and Quorum Sensing in *Chromobacterium violaceum*, *Pseudomonas aeruginosa*, and *Enterococcus faecalis* .” Microorganisms 11, no. 5: 1150. 10.3390/microorganisms11051150. 37317124 PMC10222125

[mbo370339-bib-0027] da Silva, Jr., I. I. , N. P. C. da Silva , J. A. Marrs , and P. G. Cadena . 2023. “Essential Oils Produce Developmental Toxicity in Zebrafish Embryos and Cause Behavior Changes in Zebrafish Larvae.” Biomed 11, no. 10: 2821. 10.3390/biomedicines11102821. PMC10603861 37893194

[mbo370339-bib-0028] Datta, S. , V. Singh , S. Nag , and D. N. Roy . 2025. “Carvacrol, a Monoterpenoid, Binds Quorum Sensing Proteins (LasI and LasR) and Swarming Motility Protein BswR of *Pseudomonas aeruginosa*, Resulting in Loss of Pathogenicity: An *In Silico* Approach.” Canadian Journal of Microbiology 1, no. 71: 1–15. 10.1139/cjm-2024-0155. 39566032

[mbo370339-bib-0029] Dehghani, M. M. , B. H. Naghdi , and K. M. A. Larijani . 2018. “Changes in the Essential Oil Content and Composition of *Thymus daenensis* Celak. Under Pre‐Drying and Different Storage Conditions.” Journal of Medicinal Plants 17, no. 68: 49–65. http://jmp.ir/article-1-2292-en.html.

[mbo370339-bib-0030] Deryabin, D. , A. Galadzhieva , D. Kosyan , and G. Duskaev . 2019. “Plant‐Derived Inhibitors of AHL‐Mediated Quorum Sensing in Bacteria: Modes of Action.” International Journal of Molecular Sciences 8: 5588. 10.3390/ijms20225588. PMC6888686 31717364

[mbo370339-bib-0031] Dhifi, W. , S. Bellili , S. Jazi , N. Bahloul , and W. Mnif . 2016. “Essential Oils' Chemical Characterization and Investigation of Some Biological Activities: A Critical Review.” Medicines 3: 25. 10.3390/medicines3040025. 28930135 PMC5456241

[mbo370339-bib-0032] Dodoš, T. , A. Simonović , L. Vujisić , P. D. Marin , and N. Rajčević . 2025. “Genetic Diversity and Essential Oils Variability of *Satureja subspicata* Bartl. ex Vis. Natural Populations.” Industrial Crops and Products 236: 121812. 10.1016/j.indcrop.2025.121812.

[mbo370339-bib-0033] Donadu, M. , D. Usai , A. Pinna , et al. 2018. “ *In Vitro* Activity of Hybrid Lavender Essential Oils Against Multidrug Resistant Strains of *Pseudomonas aeruginosa* .” Journal of Infection in Developing Countries 12, no. 1: 9–14. 10.3855/jidc.9920. 31628828

[mbo370339-bib-0034] Dos Santos, E. A. R. , L. E. Tadielo , J. A. Schmiedt , et al. 2023. “Effect of Ginger Essential Oil and 6‐Gingerol on a Multispecies Biofilm of *Listeria monocytogenes, Salmonella typhimurium*, and *Pseudomonas aeruginosa* .” Brazilian Journal of Microbiology 54, no. 4: 3041–3049. 10.1007/s42770-023-01075-2. 37668830 PMC10689688

[mbo370339-bib-0035] Dubois, E. , S. Gaillot , B. Valot , et al. 2025. “Adaptation to Cinnamaldehyde Shapes *Pseudomonas aeruginosa* Resistance to Major Antibiotics.” Journal of Bacteriology 207: e00180‐25. 10.1128/jb.00180-25. 41004622 PMC12548396

[mbo370339-bib-0101] ECDC . 2024. Point Prevalence Survey of Healthcare‐Associated Infections and Antimicrobial Use in European Acute Care Hospitals 2022–2023. 10.2900/88011.

[mbo370339-bib-0036] Elfadadny, A. , R. F. Ragab , M. AlHarbi , et al. 2024. “Antimicrobial Resistance of *Pseudomonas aeruginosa*: Navigating Clinical Impacts, Current Resistance Trends, and Innovations in Breaking Therapies.” Frontiers in Microbiology 5: 1374466. 10.3389/fmicb.2024.1374466. PMC11026690 38646632

[mbo370339-bib-0037] Farahpour, M. R. , E. Pirkhezr , A. Ashrafian , and A. Sonboli . 2020. “Accelerated Healing by Topical Administration of *Salvia officinalis* Essential Oil on *Pseudomonas aeruginosa* and *Staphylococcus aureus* Infected Wound Model.” Biomedicine and Pharmacotherapy 128: 110120. 10.1016/j.biopha.2020.110120. 32460189

[mbo370339-bib-0038] Ferreira de Santana, C. , I. Cirino , L. Souza , et al. 2024. “Antimicrobial and Cytotoxic Effects of *Lippia alba* Essential Oil on Multidrug‐Resistant *Acinetobacter baumannii* Clinical Isolates.” South African Journal of Botany 175: 479–485. 10.1016/j.sajb.2024.10.019.

[mbo370339-bib-0039] Figueiredo, A. C. , J. G. Barroso , L. G. Pedro , and J. J. C. Scheffer . 2008. “Factors Affecting Secondary Metabolite Production in Plants: Volatile Components and Essential Oils.” Flavour and Fragrance Journal 23: 213–226. 10.1002/ffj.1875.

[mbo370339-bib-0040] Gautam, N. , A. K. Mantha , and S. Mittal . 2014. “Essential Oils and Their Constituents as Anticancer Agents: A Mechanistic View.” BioMed Research International 2014: 154106. 10.1155/2014/154106. 25003106 PMC4070586

[mbo370339-bib-0041] Ghaderi, L. , S. Nejad Ebrahimi , and H. Rafati . 2020. “Effective Inhibition and Eradication of *Pseudomonas aeruginosa* Biofilms by *Satureja khuzistanica* Essential Oil Nanoemulsion.” Journal of Drug Delivery Science and Technology 61: 102260. 10.1016/j.jddst.2020.102260.

[mbo370339-bib-0042] Ghasemi, M. R. , A. Ranjbar , P. Tamri , S. Pourmoslemi , A. Nourian , and D. Dastan . 2023. “ *In Vitro* Antibacterial Activity and Wound Healing Effects of *Achillea millefolium* Essential Oil in Rat.” Journal of Pharmacopuncture 26, no. 2: 167–174. 10.3831/KPI.2023.26.2.167. 37405118 PMC10315885

[mbo370339-bib-0043] Gnaneswaran, T. , A. Fotouhi , K. Lynam , S. Utz , and S. Daveluy . 2025. “Essential Oils in Dermatology: This Narrative Review Evaluates the Dermatological Uses, Benefits, and Risks of Essential Oils, Providing Evidence‐Based Guidance Amid Growing Interest in Natural Healthcare Alternatives.” JOID 1, no. 1. 10.64550/joid.nt5zgk07.

[mbo370339-bib-0044] Guerriaud, M. 2018. “Regulation of Essential Oils, a Safety Imperative—Réglementation des huiles essentielles, un besoin de sécurité.” Actualités Pharmaceutiques 57: 21–25. 10.1016/j.actpha.2018.09.005.

[mbo370339-bib-0045] Hammer, K. A. , C. F. Carson , T. V. Riley , and J. B. Nielsen . 2006. “A Review of the Toxicity of *Melaleuca alternifolia* (Tea Tree) Oil.” Food and Chemical Toxicology 44, no. 5: 616–625. 10.1016/j.fct.2005.09.001. 16243420

[mbo370339-bib-0046] Haripriyan, J. , C. R. Binu , N. D. Menon , et al. 2025. “Essential Oils Modulate Virulence Phenotypes in a Multidrug‐Resistant Pyomelanogenic *Pseudomonas aeruginosa* Clinical Isolate.” Scientific Reports 30: 3738. 10.1038/s41598-025-86515-9. PMC11782693 39885214

[mbo370339-bib-0047] Helander, I. M. , H. L. Alakomi , K. Latva‐Kala , et al. 1998. “Characterization of the Action of Selected Essential Oil Components on Gram‐Negative Bacteria.” Journal of Agricultural and Food Chemistry 46: 3590–3595. 10.1021/jf980154m.

[mbo370339-bib-0048] Hinsa, S. M. , and G. A. O'Toole . 2004. “Mechanisms of Adhesion by *Pseudomonads* .” In Pseudomonas, edited by J. L. Ramos . Springer. 10.1007/978-1-4419-9086-0_23.

[mbo370339-bib-0049] Hu, F. , P. Wang , Y. Li , et al. 2023. “Bioremediation of Environmental Organic Pollutants by *Pseudomonas aeruginosa*: Mechanisms, Methods and Challenges.” Environmental Research 15: 117211. 10.1016/j.envres.2023.117211. 37778604

[mbo370339-bib-0050] Hulankova, R. 2024. “Methods for Determination of Antimicrobial Activity of Essential Oils *In Vitro*—A Review.” Plants (Basel) 13, no. 19: 2784. 10.3390/plants13192784. 39409654 PMC11478843

[mbo370339-bib-0051] Husain, F. M. , I. Ahmad , M. S. Khan , et al. 2015. “Sub‐MICs of *Mentha piperita* Essential Oil and Menthol Inhibits AHL Mediated Quorum Sensing and Biofilm of Gram‐Negative Bacteria.” Frontiers in Microbiology 6: 420. 10.3389/fmicb.2015.00420. 26029178 PMC4429619

[mbo370339-bib-0052] Huszczynski, S. M. , J. S. Lam , and C. M. Khursigara . 2020. “The Role of *Pseudomonas aeruginosa* Lipopolysaccharide in Bacterial Pathogenesis and Physiology.” Pathogens 9: 6. 10.3390/pathogens9010006. PMC7168646 31861540

[mbo370339-bib-0053] Iman Islamieh, D. , H. Goudarzi , A. Khaledi , D. Afshar , and D. Esmaeili . 2020. “Reduced Efflux Pumps Expression of *Pseudomonas aeruginosa* With *Satureja khuzistanica* Essential Oil.” Iranian Journal of Medical Sciences 45, no. 6: 463–468. 10.30476/ijms.2019.72675. 33281263 PMC7707626

[mbo370339-bib-0054] Kačániová, M. , Z. Ban , L. Li , et al. 2025. “Chemical and Biological Properties of *Elettaria cardamomum* Maton var. *minuscula* Essential Oil and Its Effect as Preservative to Shelf‐Life Storage of Sous Vide Carrot Inoculated With *Pseudomonas aeruginosa* .” Journal of Food Processing and Preservation 2025, no. 1: 7589175. 10.1155/jfpp/7589175.

[mbo370339-bib-0055] Khan, M. H. , N. A. Dar , B. A. Alie , et al. 2023. “Unraveling the Variability of Essential Oil Composition in Different Accessions of *Bunium persicum* Collected From Different Temperate Micro‐Climates.” Molecules 28: 2404. 10.3390/molecules28052404. 36903647 PMC10005284

[mbo370339-bib-0056] Khan, P. , A. Waheed , M. Azeem , et al. 2023. “Essential Oil From *Tagetes minuta* Has Antiquorum Sensing and Antibiofilm Potential Against *Pseudomonas aeruginosa* Strain PAO1.” ACS Omega 8, no. 39: 35866–35873. 10.1021/acsomega.3c03507. 37810677 PMC10551919

[mbo370339-bib-0057] Khezri, K. , M. R. Farahpour , and S. Mounesi Rad . 2019. “Accelerated Infected Wound Healing by Topical Application of Encapsulated Rosemary Essential Oil into Nanostructured Lipid Carriers.” Artificial Cells, Nanomedicine, and Biotechnology 47, no. 1: 980–988. 10.1080/21691401.2019.1582539. 30857435

[mbo370339-bib-0058] Lee, J. , and L. Zhang . 2015. “The Hierarchy Quorum Sensing Network in *Pseudomonas aeruginosa* .” Protein & Cell 6: 26–41. 10.1007/s13238-014-0100-x. 25249263 PMC4286720

[mbo370339-bib-0059] Licea‐Herrera, J. I. , A. Guerrero , M. Mireles‐Martínez , et al. 2024. “Agricultural Soil as a Reservoir of *Pseudomonas aeruginosa* With Potential Risk to Public Health.” Microorganisms 30: 2181. 10.3390/microorganisms12112181. PMC11596188 39597570

[mbo370339-bib-0060] Lu, M. , K. I. Wong , X. Li , et al. 2022. “Oregano Oil and Harmless Blue Light to Synergistically Inactivate Multidrug‐Resistant *Pseudomonas aeruginosa* .” Frontiers in Microbiology 13: 810746. 10.3389/fmicb.2022.810746. 35359746 PMC8961286

[mbo370339-bib-0061] Luciardi, M. C. , M. Amparo Blázquez , E. Cartagena , A. Bardón , and M. E. Arena . 2016. “Mandarin Essential Oils Inhibit Quorum Sensing and Virulence Factors of *Pseudomonas aeruginosa* .” LWT—Food Science and Technology 68: 373–380. 10.1016/j.lwt.2015.12.056. 31684768

[mbo370339-bib-0062] Luciardi, M. C. , M. A. Blázquez , M. R. Alberto , E. Cartagena , and M. E. Arena . 2021. “Lemon Oils Attenuate the Pathogenicity of *Pseudomonas aeruginosa* by Quorum Sensing Inhibition.” Molecules 26, no. 10: 2863. 10.3390/molecules26102863. 34066034 PMC8151035

[mbo370339-bib-0063] Marzban, A. , A. Niknejad , V. Tafakori , S. A. Feyli , and M. Karkhane . 2025. “Multifunctional Hydrogel of Zein‐Tartaric Acid Encapsulated With Oregano Essential Oils to Combat *Pseudomonas aeruginosa* Through Biofilm Inhibition.” Journal of Drug Delivery Science and Technology 108: 106934. 10.1016/j.jddst.2025.106934.

[mbo370339-bib-0064] Modarresi, M. , M. R. Farahpour , and B. Baradaran . 2019. “Topical Application of *Mentha piperita* Essential Oil Accelerates Wound Healing in Infected Mice Model.” Inflammopharm 27, no. 3: 531–537. 10.1007/s10787-018-0510-0. 29980963

[mbo370339-bib-0065] Mourabiti, F. , R. Derdak , A. Amrani , et al. 2024. “The Antimicrobial Effectiveness of *Rosmarinus officinalis*, *Lavandula angustifolia*, and *Salvia officinalis* Essential Oils Against *Klebsiella pneumoniae* and *Pseudomonas aeruginosa* In Vitro and In Silico.” South African Journal of Botany 168: 112–123. 10.1016/j.sajb.2024.03.015.

[mbo370339-bib-0066] Mulani, M. S. , E. E. Kamble , S. N. Kumkar , M. S. Tawre , and K. R. Pardesi . 2019. “Emerging Strategies to Combat ESKAPE Pathogens in the Era of Antimicrobial Resistance: A Review.” Frontiers in Microbiology 10: 539. 10.3389/fmicb.2019.00539. 30988669 PMC6452778

[mbo370339-bib-0067] Munguia, R. , J. Cadena , and S. I. Miller . 2017. “The Mla Pathway in *Pseudomonas aeruginosa* Is Critical for Maintaining Outer Membrane Integrity.” mBio 8: e00662‐17. 10.1128/mBio.00662-17.

[mbo370339-bib-0068] Nazzaro, F. , F. Fratianni , L. De Martino , R. Coppola , and V. De Feo . 2013. “Effect of Essential Oils on Pathogenic Bacteria.” Molecules 18: 12988–12999. 10.3390/molecules181212988. PMC3873673 24287491

[mbo370339-bib-0069] Neagu, R. , V. Popovici , L. E. Ionescu , et al. 2024. “Phytochemical Screening and Antibacterial Activity of Commercially Available Essential Oils Combinations With Conventional Antibiotics Against Gram‐Positive and Gram‐Negative Bacteria.” Antibiotics (Basel) 13, no. 6: 478. 10.3390/antibiotics13060478. 38927145 PMC11200707

[mbo370339-bib-0070] Noumi, E. , A. Merghni , M. M. Alreshidi , et al. 2018. “ *Chromobacterium violaceum* and *Pseudomonas aeruginosa* PAO1: Models for Evaluating Anti‐Quorum Sensing Activity of *Melaleuca alternifolia* Essential Oil and Its Main Component Terpinen‐4‐ol.” Molecules 23: 2672. 10.3390/molecules23102672. 30336602 PMC6222492

[mbo370339-bib-0071] Novelli, M. , F. Bleffert , and J. G. Hurdle . 2025. “Outer Membrane Disruption as a Strategy to Potentiate Antibiotics Against *Pseudomonas aeruginosa* .” International Journal of Molecular Sciences 26: 9844. 10.3390/ijms26209844. 41155139 PMC12563807

[mbo370339-bib-0072] Omarizadeh, K. , M. R. Farahpour , and M. Alipour . 2021. “Topical Administration of an Ointment Prepared From *Satureja sahendica* Essential Oil Accelerated Infected Full‐Thickness Wound Healing by Modulating Inflammatory Response in a Mouse Model.” Wounds 33, no. 12: 321–328. 10.25270/wnds/321328. 34882574

[mbo370339-bib-0073] Pandur, E. , G. Micalizzi , L. Mondello , A. Horváth , K. Sipos , and G. Horváth . 2022. “Antioxidant and Anti‐Inflammatory Effects of Thyme (*Thymus vulgaris* L.) Essential Oils Prepared at Different Plant Phenophases on *Pseudomonas aeruginosa* LPS‐Activated THP‐1 Macrophages.” Antioxidants 11: 1330. 10.3390/antiox11071330. 35883820 PMC9311800

[mbo370339-bib-0074] Parkins, M. D. , R. Somayaji , and V. J. Waters . 2018. “Epidemiology, Biology, and Impact of Clonal *Pseudomonas aeruginosa* Infections in Cystic Fibrosis.” Clinical Microbiology Reviews 31: e00019‐18. 10.1128/CMR.00019-18. 30158299 PMC6148191

[mbo370339-bib-0075] Pearson, J. P. , K. M. Gray , L. Passador , et al. 1994. “Structure of the Autoinducer Required for Expression of *Pseudomonas aeruginosa* Virulence Genes.” Proceedings of the National Academy of Sciences of the United States of America 91: 197–201. 10.1073/pnas.91.1.197. 8278364 PMC42913

[mbo370339-bib-0076] Pejčić, M. , Z. Stojanović‐Radić , M. Genčić , M. Dimitrijević , and N. Radulović . 2020. “Anti‐Virulence Potential of Basil and Sage Essential Oils: Inhibition of Biofilm Formation, Motility and Pyocyanin Production of *Pseudomonas aeruginosa* Isolates.” Food and Chemical Toxicology 141: 111431. 10.1016/j.fct.2020.111431. 32417365

[mbo370339-bib-0077] Perez, N. , M. J. Altube , L. R. S. Barbosa , E. L. Romero , and A. P. Perez . 2022. “ *Thymus vulgaris* Essential Oil + Tobramycin Within Nanostructured Archaeolipid Carriers: A New Approach Against *Pseudomonas aeruginosa* Biofilms.” Phytomedicine 102: 154179. 10.1016/j.phymed.2022.154179. 35671606

[mbo370339-bib-0078] Pesci, E. C. , J. B. Milbank , J. P. Pearson , et al. 1999. “Quinolone Signaling in the Cell‐To‐Cell Communication System of *Pseudomonas aeruginosa* .” Proceedings of the National Academy of Sciences of the United States of America 96: 11229–11234. 10.1073/pnas.96.20.11229. 10500159 PMC18016

[mbo370339-bib-0079] Qaralleh, H. , S. A. M. Saghir , M. O. Al‐limoun , et al. 2024. “Effect of *Matricaria aurea* Essential Oils on Biofilm Development, Virulence Factors and Quorum Sensing‐Dependent Genes of *Pseudomonas aeruginosa* .” Pharmaceuticals 17: 386. 10.3390/ph17030386. 38543172 PMC10975660

[mbo370339-bib-0080] Ramsey, J. T. , Y. Li , Y. Arao , et al. 2019. “Lavender Products Associated With Premature Thelarche and Prepubertal Gynecomastia: Case Reports and Endocrine‐Disrupting Chemical Activities.” Journal of Clinical Endocrinology and Metabolism 104, no. 11: 5393–5405. 10.1210/jc.2018-01880. 31393563 PMC6773459

[mbo370339-bib-0081] Rathinam, P. , H. S. Vijay Kumar , and P. Viswanathan . 2017. “Eugenol Exhibits Anti‐Virulence Properties by Competitively Binding to Quorum Sensing Receptors.” Biofouling 33, no. 8: 624–639. 10.1080/08927014.2017.1350655. 28792229

[mbo370339-bib-0082] Rathore, S. , and R. Kumar . 2022. “Essential Oil Content and Compositional Variability of *Lavandula* Species Cultivated in the Mid Hill Conditions of the Western Himalaya.” Molecules 27: 3391. 10.3390/molecules27113391. 35684332 PMC9182314

[mbo370339-bib-0083] Rawat, V. , S. Rawat , P. Kewlani , et al. 2025. “Essential Oil and Polyphenolic Composition in *Nardostachys jatamansi* Influenced by Different Plant Parts and Drying Conditions.” Discover Plants 2: 280. 10.1007/s44372-025-00360-8.

[mbo370339-bib-0084] Razdan, K. , S. Kanta , E. Chaudhary , et al. 2023. “Levofloxacin Loaded Clove Oil Nanoscale Emulgel Promotes Wound Healing in *Pseudomonas aeruginosa* Biofilm Infected Burn Wound in Mice.” Colloids and Surfaces B: Biointerfaces 222: 113113. 10.1016/j.colsurfb.2022.113113. 36566688

[mbo370339-bib-0085] Roman, H. , A.‐G. Niculescu , V. Lazăr , and M. M. Mitache . 2023. “Antibacterial Efficiency of *Tanacetum vulgare* Essential Oil Against ESKAPE Pathogens and Synergisms With Antibiotics.” Antibiotics (USSR) 12: 1635. 10.3390/antibiotics12111635. PMC10669310 37998837

[mbo370339-bib-0086] Sena, G. , E. De Rose , M. Crudo , et al. 2024. “Essential Oils From Southern Italian Aromatic Plants Synergize With Antibiotics Against *Escherichia coli*, *Pseudomonas aeruginosa* and *Enterococcus faecalis* Cell Growth and Biofilm Formation.” Antibiotics (USSR) 13: 605. 10.3390/antibiotics13070605. PMC11274178 39061287

[mbo370339-bib-0087] Siddique, A. B. , H. Ahsan , M. Shahid , et al. 2025. “Preparation and Characterization of Essential Oil From *Lavandula spica* Plant and Its Antimicrobial Activity Against *Pseudomonas aeruginosa* and *Staphylococcus aureus* .” Microbial Pathogenesis 198: 107157. 10.1016/j.micpath.2024.107157. 39603567

[mbo370339-bib-0088] Simbu, S. , A. Orchard , and S. van Vuuren . 2024. “Essential Oil Compounds in Combination With Conventional Antibiotics for Dermatology.” Molecules 29: 1225. 10.3390/molecules29061225. 38542862 PMC10974782

[mbo370339-bib-0089] Sœur, J. , L. Marrot , P. Perez , et al. 2011. “Selective Cytotoxicity of *Aniba rosaeodora* Essential Oil Towards Epidermoid Cancer Cells Through Induction of Apoptosis.” Mutation Research/DNA Repair 718, no. 1–2: 24–32. 10.1016/j.mrgentox.2010.10.009. 21070863

[mbo370339-bib-0090] Srivastava, A. , V. Kumar , D. Sharma , and V. Agarwal . 2023. “Data Regarding Anti‐Quorum Sensing and Antimicrobial Activity of *Melaleuca alternifolia* and *Salvia sclarea* Essential Oil Against *Pseudomonas aeruginosa* .” Data in Brief 48: 109145. 10.1016/j.dib.2023.109145. 37383790 PMC10293969

[mbo370339-bib-0091] Suresh, D. , S. VijayaKumar , P. Pradhan , and S. Balasubramanian . 2025. “Fatal Eucalyptus Oil Poisoning in an Adult Male: A Case Report With Comprehensive Autopsy and Histopathological Findings.” Cureus 17, no. 4: e83053. 10.7759/cureus.83053. 40432665 PMC12107212

[mbo370339-bib-0092] Tapia‐Rodriguez, M. R. , A. T. Bernal‐Mercado , M. M. Gutierrez‐Pacheco , et al. 2019. “Virulence of *Pseudomonas aeruginosa* Exposed to Carvacrol: Alterations of the Quorum Sensing at Enzymatic and Gene Levels.” Journal of Cell Communication and Signaling 13, no. 4: 531–537. 10.1007/s12079-019-00516-8. 30903602 PMC6946780

[mbo370339-bib-0093] Tetard, A. , S. Gaillot , E. Dubois , et al. 2022. “Exposure of *Pseudomonas aeruginosa* to Cinnamaldehyde Selects Multidrug Resistant Mutants.” Antibiotics (USSR) 11: 1790. 10.3390/antibiotics11121790. PMC9774640 36551447

[mbo370339-bib-0094] Thi, M. T. T. , D. Wibowo , and B. H. A. Rehm . 2020. “ *Pseudomonas aeruginosa* Biofilms.” International Journal of Molecular Sciences 17: 8671. 10.3390/ijms21228671. PMC7698413 33212950

[mbo370339-bib-0095] Ultee, A. , E. P. W. Kets , and E. J. Smid . 2002. “Mechanisms of Action of Carvacrol on the Food‐Borne Pathogen *Bacillus cereus* .” Applied and Environmental Microbiology 65: 4606–4610. 10.1128/AEM.65.10.4606-4610.2002. PMC91614 10508096

[mbo370339-bib-0096] Van, L. T. , I. Hagiu , A. Popovici , et al. 2022. “Antimicrobial Efficiency of Some Essential Oils in Antibiotic‐Resistant *Pseudomonas aeruginosa* Isolates.” Plants 11: 2003. 10.3390/plants11152003. 35956481 PMC9370326

[mbo370339-bib-0097] Vrenna, G. , M. Artini , R. Ragno , et al. 2021. “Anti‐Virulence Properties of *Coridothymus capitatus* Essential Oil Against *Pseudomonas aeruginosa* Clinical Isolates From Cystic Fibrosis Patients.” Microorganisms 9, no. 11: 2257. 10.3390/microorganisms9112257. 34835383 PMC8623622

[mbo370339-bib-0098] Wang, X.‐Y. , J.‐M. Hao , Q.‐R. Ren , et al. 2021. “Cytotoxicity and Apoptosis Induced by *Chenopodium ambrosioides* L. Essential Oil in Human Normal Liver Cell Line L02 via the Endogenous Mitochondrial Pathway Rather Than the Endoplasmic Reticulum Stress.” International Journal of Environmental Research and Public Health 18: 7469. 10.3390/ijerph18147469. 34299918 PMC8304090

[mbo370339-bib-0099] Williamson, E. M. 2001. “Synergy and Other Interactions in Phytomedicines.” Phytomed 8, no. 5: 401–409. 10.1078/0944-7113-00060. 11695885

[mbo370339-bib-0100] Zawadzińska, A. , A. Wesołowska , E. Skutnik , J. Rabiza‐Świder , and P. Salachna . 2025. “Changes in Growth and Chemical Composition of the Essential Oil From Flowers and Leafy Stems of *Lavandula angustifolia* Grown in Media Amended With Bark and Sewage Sludge.” Molecules 30: 4545. 10.3390/molecules30234545. 41375142 PMC12693513

